# Neural Population Dynamics and Cognitive Function

**DOI:** 10.3389/fnhum.2020.00050

**Published:** 2020-03-12

**Authors:** Stephen E. Nadeau

**Affiliations:** Research Service and the Brain Rehabilitation Research Center, Malcom Randall VA Medical Center, Department of Neurology, College of Medicine, University of Florida, Gainesville, FL, United States

**Keywords:** parallel distributed processing, cognitive function, knowledge, memory, language, attention, executive function, emotional function

## Abstract

Representations in the brain are encoded as patterns of activity of large populations of neurons. The science of population encoded representations, also known as parallel distributed processing (PDP), achieves neurological verisimilitude and has been able to account for a large number of cognitive phenomena in normal people, including reaction times (and reading latencies), stimulus recognition, the effect of stimulus salience on attention, perceptual invariance, simultaneous egocentric and allocentric visual processing, top-down/bottom-up processing, language errors, the effect of statistical regularities of experience, frequency, and age of acquisition, instantiation of rules and symbols, content addressable memory and the capacity for pattern completion, preservation of function in the face of noisy or distorted input, inference, parallel constraint satisfaction, the binding problem and gamma coherence, principles of hippocampal function, the location of knowledge in the brain, limitations in the scope and depth of knowledge acquired through experience, and Piagetian stages of cognitive development. PDP studies have been able to provide a coherent account for impairment in a variety of language functions resulting from stroke or dementia in a large number of languages and the phenomenon of graceful degradation observed in such studies. They have also made important contributions to our understanding of attention (including hemispatial neglect), emotional function, executive function, motor planning, visual processing, decision making, and neuroeconomics. The relationship of neural network population dynamics to electroencephalographic rhythms is starting to emerge. Nevertheless, PDP approaches have scarcely penetrated major areas of study of cognition, including neuropsychology and cognitive neuropsychology, as well as much of cognitive psychology. This article attempts to provide an overview of PDP principles and applications that addresses a broader audience.

## Introduction

In 1980, it was not possible to imagine how a brain composed of 100 billion highly interconnected, lipid-encased, reticular electrochemical devices could possibly support complex neural functions like language, memory, visuospatial, emotional and executive function. However, thanks to the epochal two-volume work of McClelland et al. (1986) on parallel distributed processing (PDP; McClelland et al., 1986), the vast outpouring of research they have conducted and inspired since then on population encoded (distributed) representations (reviewed on 25th anniversary of PDP by Rogers and McClelland, 2014), and the parallel advances in our understanding of the corresponding processes at the neural level (Rolls, 2016), we now have a remarkably detailed understanding of the relationships between neural structure and higher neural functions. We understand that the brain provides the essential network scaffold for cognitive processing but that the substance of that processing is acquired through a lifetime of learning and that the rules governing that processing are implicit and emergent and reflect the statistical properties of experience (Elman et al., 1996; Plaut and Vande Velde, 2017). The precise details of the scaffold may differ from person to person, perhaps reflecting, in part, individual differences in cytoarchitectonic maps (Rajkowska and Goldman-Rakic, 1995) and white matter connectivity (most conspicuous in synesthesia), as well as underlying genetic and evolving epigenetic influences, but the general features are likely common to all human brains.

Here, I review the major insights that PDP research has provided into the neural basis of cognitive function. Much of this science is now fairly mature but, as with any good theory, domains of uncertainty benefit from the coherence of the theoretical components that have emerged and the extensive empirical validation of PDP theory. PDP theory can now account for an enormous spectrum of psychophysical and behavioral phenomena without the benefit of *ad* *hoc* or algorithmic devices. Maturity, coherence, empirical validation, and neural verisimilitude inspire confidence that the account to be elaborated here is basically correct. In the interests of clarity, the story will be related with little qualification and reliance on the reader to know that this remains a theory.

Even now, nearly 35 years after the publication of McClelland et al. (1986), neither the importance of the neural verisimilitude of PDP models nor their enormous implicit explanatory power for cognitive and behavioral function in health and brain disease have been widely recognized beyond the PDP community. In the conclusion to this article, I will consider why this might be so.

## The Structure of Population Encoding Networks

It has been known for some time that representations in the central nervous system (CNS) are population encoded, that is, encoded as patterns of activity involving very large numbers of highly interconnected neurons in one or more neural networks extending over large expanses of the brain (O’Keefe and Nadel, 1979; Georgopoulos et al., 1982; Churchland and Sejnowski, 1992; Rolls and Treves, 1998; Zhang et al., 1998; Zhang and Sejnowski, 1999; Rolls and Deco, 2002; Behrmann and Plaut, 2013; Rolls, 2016; Lebedev and Nicolelis, 2017). The properties of population encoding networks have been extensively explored in simulations. Most of these involve fairly simple mathematics: unit activations between 0 and 1 defined as a sigmoid (i.e., logistic) function (∫), reflecting existing activation levels and input from all afferent units, each afferent unit input multiplied by the weight of its connection (corresponding roughly to synaptic strengths in the brain), and output defined as a nonlinear function of unit activation, often incorporating a “firing” threshold. To one degree or another, units are highly and reciprocally interconnected (hence the term “connectionist model”), as in the brain (Felleman and Van Essen, 1991), and activity is understood to flow between units throughout a network and all connected networks. Knowledge is represented in connection strengths and learning consists of alterations of connection strengths. These simple mathematics obviously do not do justice to all the subtleties of actual neural processing but they do capture the most essential properties of neural activity and interactivity. For this reason, they have given us powerful insights into brain function and they have been extraordinarily successful in predicting behavior in normal and brain-damaged individuals. The implicit properties of networks employing these mathematics, to be detailed below, provide an orderly explanation for a host of brain functions and dysfunctions.

For computational models employing population encoding to provide useful insights into brain function, they must represent hypotheses that transparently respect neural verisimilitude. *Ad hoc* structures or algorithmic appendages will likely detract from such verisimilitude. Query of the internal processes of the model, e.g., assessment of hidden unit activity in particular model states, can elucidate processes that may be occurring in the brain.

Two major types of networks have been defined: auto-associator and pattern associator. In auto-associator networks, units are substantially interconnected with each other, giving the networks attractor properties: the capacity for settling within an attractor basin into an attractor state that is optimal, or at least quasi-optimal (more about this later), given the pattern of inputs to the network. Pattern associator networks translate patterns of activity in one representational domain (e.g., orthographic input) into another representational domain (e.g., semantic representations or articulatory representations). Pattern associator networks commonly incorporate “hidden units” between the input and output layers. Hidden units, combined with the nonlinear properties of all units in the network, enable such things as translation between largely orthogonal domains (e.g., between word meaning and word sound) and the incorporation of sequence knowledge. Hidden units support representations that are not directly definable in behavioral terms. The entire brain can be viewed as a vast ensemble of hidden units and only at the inputs (e.g., retinal ganglion cells) and outputs (motor neurons), can the unit activity be directly mapped to the environment or to observable behavior. The function of hidden units must be inferred from their connectivity patterns, observable subject behaviors, and computer simulations.

**Figure 1:**
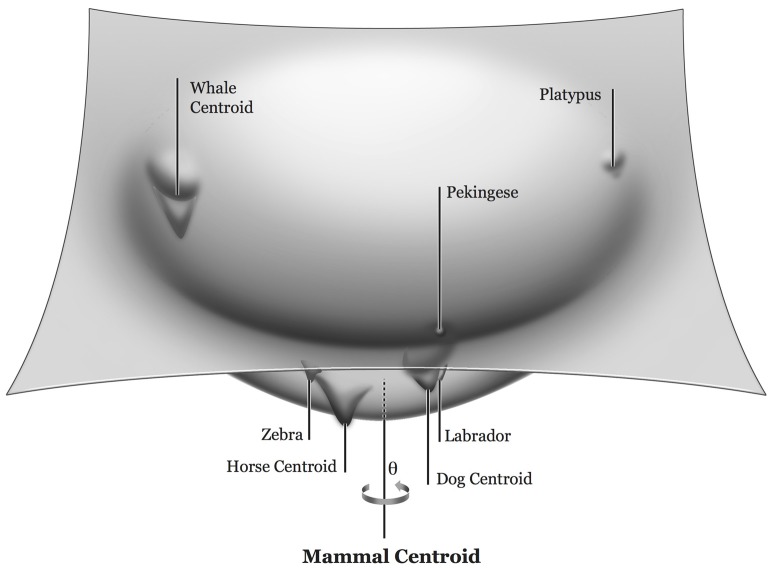
The topography of the semantic network energy function in the vicinity of the mammal attractor basin. Each point corresponds to an energy level of all features in an N-dimensional feature hyperspace. The point of maximal typicality is represented by the centroid of a basin/sub-basin. Distance from the centroid reflects the degree of atypicality. The value of θ defines the manner in which atypicality is defined. For example, whales and platypuses are both atypical but in very different ways. From Nadeau (2012), with permission.

We can give these abstract principles substance with an illustration from semantics. Our knowledge of the world and the objects within it is encoded in association cortices throughout the brain. A semantic representation, e.g., of a dog, corresponds to a locus in an N-dimensional neural activity feature space. Figure 1 illustrates the results of a thought experiment that involves taking a 3-dimensional slice of the corresponding energy landscape, a slab in the vicinity of mammal knowledge. The central, lowest “energy” point—the “centroid” of mammal knowledge—corresponds to the representation of a creature that best defines our sense of mammalness. Within the mammal basin, there are innumerable attractor sub-basins corresponding to specific mammals. A sub-basin is defined by the addition of features (additional neural connectivity) to exemplars of the domain to which it belongs. For example, distinguishing a dog from other mammals, or a Labrador from other dogs requires some additional feature knowledge. Very close to the centroid are sub-basins corresponding to mammals likely to be very close to the centroid representation, e.g., dogs, cats, cows, and horses. Distance from the centroid is defined by the degree of atypicality, which reflects feature and feature combination frequency within the mammal domain. Highly atypical animals, such as whales and platypuses, are represented near the periphery of this mammal attractor basin. Within any given sub-basin, there may be sub-sub-basins, for example, corresponding to types of dogs. The depth of the mammal basin and its sub-basins (the z-axis in Figure 1) is determined by the depth of the encoding of knowledge in neural connectivity. This, in turn, is determined by the degree to which a given exemplar shares features with other exemplars in the domain (corresponding to regularities and defining the depth of the “parent” basin or sub-basin), the number of unique features (defining the depth of the “daughter” sub-basin or sub-sub-basin), the frequency of the exemplar in the individual’s experience, and the age of knowledge acquisition. The network’s settled activity state is most strongly influenced by the specific input, which in most circumstances will absolutely define an attractor state within the sub-basin or sub-sub-basin into which the network settles, all the other factors exerting their major influence either on response latencies or the occasional production of errors. Errors will consist of slips into nearby sub-basins or settling into the larger parent basin. With network damage, focal or diffuse—hence loss of neural connections defining more specific features—deep basins will become shallower and sub-basins, particularly those that are shallower and more distant from the centroid—corresponding to more atypical exemplars—will disappear. As sub-basins become shallow or disappear, responses will reflect the settling of the network into surviving neighbors located nearer the centroid—neighbors of higher typicality (yielding coordinate errors, e.g., horse in lieu of donkey), the parent basin (yielding superordinate errors, e.g., animal in lieu of donkey), or failure to settle at all, yielding omission errors. This is precisely what has been observed in patients with semantic dementia (Woollams et al., 2008) and in PDP simulations of semantic dementia (Rogers and McClelland, 2004; Rogers et al., 2004).

We can extend the general idea of attractor basins generated by auto-associator networks to attractor trenches generated by pattern associator networks. An attractor trench is a translation pathway between two auto-associator domains (Nadeau, 2012, 2014). Thus, in the orthographic-phonologic network that supports reading aloud and incorporates knowledge of the relationships between sequences of letters and sequences of articulated phonemes, there is an attractor trench corresponding to “ust” words (must, bust, trust, lust, etc.; Seidenberg and McClelland, 1989; Plaut et al., 1996). There is also an attractor trench corresponding to “int” words. However, it has two sub-trenches, one corresponding to /Int/, e.g., mint, tint, flint, and lint, the other to the single /int/ word, pint. Our understanding of the attractor trench landscape of phonologic sequence knowledge has also recently advanced in corresponding ways (Vitevitch and Castro, 2015; Vitevitch and Luce, 2016).

**Figure 2:**
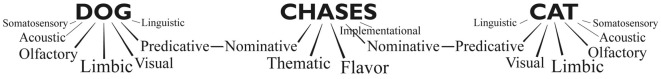
The multifocal distributed representation of a sentence. The multi-regional distribution of noun knowledge (a neural ensemble) is discussed in the section on the structure of population encoding networks. Verbs have an analogous multi-regional distributed representation, including frontal components involved in the incorporation of thematic role(s), post-central components instantiating verb flavor (manner, path, and limbic representation), an implementational component in motor cortex instantiating movement, and a nominal component corresponding to linked noun representations. From Nadeau (2012), with permission.

Any given entity may be represented in a number of different, linked, neural networks that only, in aggregate, support the N-dimensional manifold described above and in Figure 1. This general idea dates back to Lissauer (1988) and Wernicke (Eggert, 1977). It has recently been captured by the term neural ensembles (Pulvermüller, 2010). Thus, the concept of a dog has a visual representational component in visual association cortices (what dogs in general or particular dogs look like), a somatosensory component (soft fur, cold nose), a limbic component (what we feel about dogs in general or our own pets), an acoustic component, an olfactory component, a predicative component (what dogs commonly do or have done to them; how objects can be manipulated), and linguistic components (Nadeau, 2012; Figure 2). Thus, a stable cerebral representation of dog actually corresponds to an entire constellation of attractor states in interconnected attractor basins. In this view, much of the cortical surface will be engaged by a concept representation. Damage to limited regions of the brain will be reflected in deficits in particular components of meaning. For example, English speaking patients with posterior left temporal lesions retain a reasonable ability to use verbs (Breedin et al., 1994; Breedin and Martin, 1996; Marshall et al., 1996; Nadeau, 2012). However, they have difficulty distinguishing between words like walk, trudge, saunter, strut, march, sashay, stroll and pace, reflecting loss of knowledge of the manner component of verb meaning.

If a domain of concepts is particularly dependent upon one neural network in an ensemble, damage to that network may produce differential impairment. For example, damage to the visual association cortex, e.g., by herpes simplex encephalitis, results in category-specific deficits in naming and recognition for living things because the visual component of living things constitutes an indispensable component of our knowledge of them (Warrington and Shallice, 1984; Forde and Humphreys, 1999). Farah and McClelland (1991), in a PDP simulation, inquired into the essential nature of the interrelationship between components of a neural ensemble comprised of two domains, visual (providing the principal contribution to knowledge of living things) and functional (providing the principal contribution to knowledge of nonliving things, e.g., tools).

A large number of components of neural ensembles have implications for multi-tasking because it creates a high probability (higher than typically considered Feng et al., 2014) that two different ensemble representations will engage the same region of cortex, thus competing. For example, language tasks involving movement verbs interfere with movement because both engage precentral gyrus (see Nadeau, 2012). Multi-tasking may also be constrained by limited ability to simultaneously maintain two volitional plan representations.

## Functional Implications of Population Encoding Networks

Neural networks incorporating these simple characteristics have a number of implicit properties that are directly relevant to brain-behavior relationships. Understanding these properties enables one to understand essentially any domain of a cognitive function in entirely new and very productive ways even without doing computer simulations.

1. Processing occurs and knowledge (long-term memories) is stored (as synaptic strengths) in exactly the same network. For example, visual association cortices both process visual input and store visual knowledge. Dominant (and to a lesser and variable extent, non-dominant) perisylvian cortex stores knowledge of phonological sequences and supports phonological processing. The fact that networks encoding knowledge also support processing enables such things as stimulus recognition (or sense of familiarity) and reactive attention driven by stimulus salience, familiarity, or context (Spratling and Johnson, 2004). Concurrent encoding of knowledge and support for processing also enables simultaneous visual viewer-centered (egocentric and stimulus-derived) and object-centered (allocentric and knowledge derived) processing of visual stimuli (Mozer, 2002).

Working memory and attention are subserved by essentially the same process of selective engagement: the bringing on line of selected representations in selected neural networks by eliciting alterations in the pattern of neural activity, alterations in the likelihood of neural firing, or selection of inputs that induce neural firing (Moran and Desimone, 1985; Desimone and Duncan, 1995; Nadeau and Crosson, 1997). Working memory and attention appear to correspond to deepening of attractor basins (alterations in population patterns of firing) elicited by either salient sensory input (reactive attention to particularly salient stimuli), input from prefrontal cortex corresponding to volitional attention (Rolls and Deco, 2015), or input from parietal cortex prioritizing objects at particular locations (Spratling and Johnson, 2004; Rolls, 2016). This might be accomplished through the achievement of greater synchronicity between gamma frequencies (greater coherence) in the post-central neural networks encompassed by the attractor basins (Fries, 2015; see below).

2. Capacity for settling into attractor states. The mathematical properties of neurons and the networks to which they belong assure that they will naturally settle into attractor states. As perhaps first suggested by Plaut et al. (1996), the time to settle corresponds to reaction time. The large extent of cortex engaged in these states has been nicely demonstrated in magnetoencephalographic studies (Boulenger et al., 2012; Carreiras et al., 2015; Miozzo et al., 2015; Hultén et al., 2019). Porter and Lemon (1993) were possibly the first to recognize the neural instantiation of settling in their studies of the corticospinal system. Studies of nonhuman primates had demonstrated that, whereas a cortical Betz cell could be driven to fire an anterior horn cell with a latency of about 5 ms, with naturally occurring movement, the time from Betz cell firing to anterior horn cell firing was about 100 ms. Thus, 95% of the anterior horn cell firing latency was taken up by a settling process involving linked cortical networks, the basal ganglia, the cerebellum, pontine, medullary and vestibulospinal systems, and the segmental pool of anterior horn cells. In the cerebrum, the conduction velocity of myelinated axons is likely on the order of 50 m/s. It would take an action potential about 2 ms to travel from a frontal pole to the posterior temporal or parietal cortex. Behavioral responses generated by the cerebrum take on the order of hundreds of ms. Thus, the full cerebral settling process, even if it involves distant neural networks, must subsume hundreds of back-and-forth transmissions.

PDP models are sometimes criticized for the enormous number of epochs required to train them using such standard training algorithms as backpropagation, a purely heuristic technique, as well as on their seeming dependence on the backpropagation algorithm. A typical night of sleep could afford on the order of 10^5^ volleys between any two regions (assuming entrainment to theta frequencies). If we can also assume that each volley is associated with a minor adjustment of neural connectivity in the process of memory consolidation (see below), then the scale of synaptic tweaking is comparable to that employed in PDP modeling. Backpropagation is an algorithm for network training in which the actual output is compared with the target output and then each connection is adjusted to the extent that it is contributing to error. While backpropagation as conventionally employed in PDP modeling is not biologically plausible, learning based on local rules, e.g., Hebbian learning reflects not only bottom-up input patterns but also the top-down influences of connected networks.

The full representation of a complex cerebrally instantiated entity, e.g., “dog,” requires parallel settling into the constellation of linked attractor basins constituting a neural ensemble. Settling within even one attractor basin requires the reconciliation of competing influences on the pattern of neural activity and the ultimate state of the network reflects the process of parallel constraint satisfaction (McClelland et al., 2014). Simultaneous settling within a constellation of attractor basins takes parallel constraint satisfaction to an entirely new level and it is possible that it is only achieved by stages. Parallel constraint satisfaction does not assure that all constraints are fully met. Thus, the ultimately settled state is often quasi-optimal, rather than optimal. Hence our propensity for making syntactic gaffs, occasional semantic paraphasias, phonological slips, and even anomia, the latter reflecting an inability to settle into any phonological attractor state. These problems are magnified in the context of brain damage.

The settling process subsumes bottom-up/top-down processing. Because of bottom-up/top-down processing, what we perceive actually corresponds to the outcome of a negotiation between networks that are the direct recipient of sensory input and networks supporting our knowledge of the world and what we plan to do with it (Carreiras et al., 2014). Hence the editor’s curse: we overlook typographical errors because we “see” what was intended, not what was actually written.

3. Capacity for incorporating statistical regularities of experience, frequency, and age of acquisition effects (Kumaran et al., 2016). Each addition of knowledge or skill to the brain is coded as an adjustment of neural connection strengths. Population encoding networks have been shown to be highly proficient at capturing statistical regularities in these experiences. For example, the English verbs that form a regular past tense are individually infrequent but, because they all share the same pattern of past tense formation, they avail themselves of the implicit regular past tense rule that has been instantiated in morphologic sequence connectivity through accumulated experience (Nadeau, 2012). In contrast, the past tense “rule” for irregular past tense verbs (e.g., swim—swam; hit—hit; go—went) is only somewhat reflective of regularities in morphologic sequence knowledge (because few verbs share these regularities), and knowledge of these forms is substantially reliant on the impact of frequency effects on the encoding of this type of verb past tense form. The 160 verbs with an irregular past tense are among the most frequently used in the English language. Knowledge gained early in life is more resistant to degradation in the face of brain injury than knowledge acquired later in life—the age of acquisition (AOA) effect (Rogers et al., 2004).

The AOA effect appears to be related to the gradual evolution of neural connection strengths from a normal distribution to one of extremes (high or low), an evolution that progressively limits the magnitude of further synaptic modification that can occur in association with the acquisition of new knowledge (Ellis and Lambon Ralph, 2000; Lambon Ralph and Ehsan, 2006). The mechanism of the AOA effect is counteracted by mechanisms underlying synaptic homeostasis (Tononi and Cirelli, 2014). Learning during wakefulness corresponds to increases or decreases in synaptic strengths within neural systems implicated in learning experiences. Eventually, this will lead to saturation of neural connectivity as, over time, synaptic strengths are driven to maximal or minimal values (Ellis and Lambon Ralph, 2000; Lambon Ralph and Ehsan, 2006). Not only does this steadily reduce learning capacity but it also decreases the ability to selectively encode more important memories. The synaptic homeostasis hypothesis is that during wakefulness, there is, in aggregate, an overall strengthening of synaptic connectivity, while during non-REM sleep, there occurs a “normalization” of synaptic connectivity characterized by comprehensive downgrading of synaptic connections strengths, constrained by a “survival of the fittest” process in which neural connectivity that is most implicated in the day’s knowledge acquisition and implicated in existing long-term memory will be least weakened, or even strengthened, while neural connectivity that does not share these attributes will be differentially weakened. Thus, both the capacity for further learning (neuroplasticity) and capacity for prioritization of knowledge to be retained are preserved.

The capacity to incorporate statistical regularities of experiences differs considerably between knowledge domains. It is high in the domain of semantic knowledge, albeit greater for living things than for artifacts. It is also high in pattern associator networks in which there is a high degree of correspondence between representations in one domain and representations in the other, for example, orthographic sequence knowledge and phonologic sequence knowledge. It is low in pattern associator networks linking substantially orthogonal domains, e.g., semantic knowledge and phonological sequence knowledge (there is generally little relationship between word meaning and word sound). The representation of low-frequency entities in highly regular domains is strongly supported by the features they share with other members of the domain. Exemplars in domains marked by few regularities must rely on frequency and age of acquisition for the strength of representation. Age of acquisition effects tends to be extinguished as regularities are instantiated so they are only apparent in irregular domains or in regions of irregularity in domains largely characterized by regularity (Lambon Ralph and Ehsan, 2006). Domain regularity is particularly relevant to rehabilitation because it provides a basis for generalization of gains achieved during training to performance on untrained material (Nadeau, 2015).

4. Rules and symbols. Neural architectures supporting population encoding have been criticized over the years for a perceived inability to instantiate rules and symbols. However, it should now be clear that, because the nonlinear properties of neurons and neural networks provide a basis for settling into states, whether in attractor basins or attractor trenches, this criticism is not well-founded. The creature you just saw was either a cat or a dog, not some blend of the two. The past tense of regular English verbs is formed according to an implicit rule: add /t/ (dip/dipt), /d/ (film/filmed), or /ed/ (abscond/absconded), one that corresponds to a regularity in phonologic and morphologic sequence knowledge. A rule is a sign of an attractor trench and a symbol a sign of an attractor basin.

5. Content addressable memory. Because knowledge is distributed throughout feature space, engagement of individual features can elicit entire concept representations (i.e., pattern completion). For example, the perception of a feather can elicit a population encoded representation of birds. The capacity for pattern completion is essential to perceptual invariance, the ability to recognize an object from different points of view (Mozer, 2002; Prevete et al., 2008). The same fundamental mechanism, operating instead in the dorsal “where” visual system, may provide the basis for relating retinotopic space to egocentric space. The facility for content addressable memory enables the elicitation of correct representations by corrupted input (Tang et al., 2018)—an essential capacity given the frequency with which an organism operates under conditions of degraded perception. A novel input may elicit recall of a similar pattern from memory, thereby instantiating generalization (Haberly, 2001).

6. Graceful degradation. Because knowledge is represented as synaptic connection strengths throughout a network, degradation of connections in the network will not halt function. Rather, network output will become more errorful, yielding near-miss errors or even non-responses when the network is intermittently incapable of eliciting a particular representation in an output network productive of behavior. Residual productivity will be proportionate to the strength of encoding of particular knowledge in neural network connectivity. The same principle will generally apply when there is focal damage to a network. However, because networks are not isolated and, as they merge into each other, there tends to be a gradation of function, the effects of focal and diffuse damage may not be quite the same. The behavior of population encoding networks is intrinsically stochastic; this is another contributor to the quasi-optimality of attractor states as well as to the production of fluctuating responses.

There is an additional contributor to graceful degradation. Because a concept or concept component corresponds to a pattern of activity across a multi-component, multifocal neural ensemble, there may be substantial preservation of function despite damage to one or more constituents of that ensemble, hence some preservation of comprehension of tools in the face of severe damage to visual association cortex in herpes simplex encephalitis (Farah and McClelland, 1991). It is because of graceful degradation that detailed studies of cognition in the context of brain damage can be so revealing about fundamental network properties and processes.

7. Inference. Networks may make small inferential errors when the states into which they settle are not optimal. They may make larger inferential errors if deprived of important contextual information. Thus, one might infer that the presence of a stove, refrigerator, and dishwasher signals a kitchen if unaware that the context is a department store. However, the capacity for inference that is intrinsic to population encoding networks, further empowered by the vast constellation of linked networks in the brain, confers some very powerful capabilities. It enables us to make sense of fragmentary perceptual input. It enables us to do thought experiments, arbitrarily selecting one or more features and ascertaining what sorts of distributed representations they elicit—the essence of a hypothesis. It enables us to establish constructive relationships between seemingly orthogonal constructions, for example, the N-dimensional mathematics of semantic feature hyperspace, a visual surface in 3-dimensions, and the signs of semantic dementia, as discussed in the foregoing.

These considerations, in aggregate, depict a picture of brain function that is substantially at odds with conceptualizations that are common these days.

First, although innumerable focal processes can be identified, generally defined by the somewhat opaque neurodynamics defined by hidden unit processing and settling into attractor basins and trenches, even seemingly simple processes like naming a picture engage much of the brain.

Second, the order that emerges is a chaotic order (Gleick, 1987), the order that emerges from the activity of billions of heavily interactive units, each expressing a limited spectrum of functional parameters.

Third, brain states defining observable behavior reflect a settling process involving hundreds of back and forth volleys between participating neural networks.

Fourth, a great deal of processing occurs automatically in this settling process with its reconciliation of activity patterns in different autoassociator networks *via* pattern associator networks (parallel constraint satisfaction).

What is not automatic is largely the province of the frontal lobes: volitional planning, volitional decision making, the volitional engagement of select neural networks that defines the processes of working memory and volitional attention, and the volitional sequencing and modification of distributed concept representations in the processes of thinking and speaking (syntax; Nadeau, 2012). Automatic and volition processes correspond, respectively, to the “fast” and “slow” of Daniel Kahneman’s landmark book, “Thinking: Fast and Slow” (Kahneman, 2011). Kahneman’s work, which takes into account a vast psychological literature, explores at length the intrinsic strengths, weaknesses, and proclivities of the two processes, the fluctuating balance between the two that occurs in natural behavioral contexts, not always to advantage, and the ways in which that balance can be manipulated under experimental control. Reactive processes, based predominantly in postcentral cortices, bring to bear the powerful associational and inferential capabilities born of PDP. However, they are prone to error when the knowledge base for inference is too small, based on stereotypes, or inherently unpredictable, or the correct thought, decision or action requires the application of algorithmic processes, particularly those taking into account such statistical phenomena as base rate effects, regression to the mean, and the inverse association between variance and sampled population size. In this conceptualization, creativity (Heilman et al., 2003; Heilman, 2005) is based on an iterative dialogue between reactive and volitional systems.

Much has been written about the binding problem: the capacity for linking neural representations in various parts of the brain. Population encoding networks with the properties described should solve the binding problem through their capacity for settling into constellations of attractor states, in the process achieving parallel constraint satisfaction. However, for this to happen, the hundreds of back and forth volleys of neural transmission comprising the settling process must be precisely synchronized, else they will only contribute noise. This has been the focus of intense study and this field, still rapidly emerging, has proven to be very complex (Fries, 2015). For effective transmission to occur between neurons in any two post-central networks, their gamma frequency (30–90 Hz) oscillations must be similar (i.e., there must be coherence). In this way, transmissions from one network will arrive at the other during the optimal temporal point of neuronal excitation, rather than during the period of post-excitatory inhibition, when they will have less if any, effect. Gamma-synchronization is modulated by frontal input in the alpha-beta (8–20 Hz) frequency range. Thus, frontal input serves to achieve relatively greater coherence in networks engaged in the processing of attended stimuli. In this conceptualization, control is by 8–20 Hz frequencies emanating from frontal and parietal regions while implementation is achieved through coherence of post-central 30–90 Hz frequencies shared by engaged post-central networks. On the other hand, attentional mechanisms are entrained to sample stimuli competing for attention at 7–8 Hz theta rhythms. Working memory impairment observed in elderly subjects correlates with theta phase/gamma amplitude de-coupling between the dorsolateral prefrontal cortex and the lateral temporal cortex and can be normalized by individualized theta rhythm transcranial alternating current stimulation of these areas (Reinhart and Nguyen, 2019). It is possible that the different rates of oscillations observed in the cerebral cortex (e.g., alpha, theta, beta, gamma), and their fluctuations over time, reflect the distinctive properties of the neural networks in the regions involved, and serve to synchronize or desynchronize connectivity within and between different portions of the cortex according to cerebral processing demands.

One very important implication of these discoveries on the neural mechanisms of binding is that functional connectivity derived from functional imaging studies is state-specific. Anatomically connected networks with high gamma coherence will exhibit high functional connectivity and those with low gamma coherence low functional connectivity. This has long been suspected. For example, the motor cortex can be engaged in performing movements or by the implementational component of the neural representation of movement verbs (Figure 2), depending on the circumstances (Nadeau, 2012).

Before our brief review of PDP studies of cognitive functions in all their diversity, four essential questions need to be addressed.

First, the dense patterns of cortical interconnectivity that are responsible for many of the most powerful attributes of cortical function and that support extensively overlapping representations (reviewed above) are fundamentally incompatible with the process of rapid acquisition of new declarative knowledge as episodic memories. Most critically, rapidly acquired new knowledge in such networks will replace existing knowledge, a phenomenon known as catastrophic interference (McCloskey and Cohen, 1989; McClelland et al., 1995). In addition, new knowledge must be linked to established cortical knowledge relevant to the specific experience to be remembered and not to larger, more general domains of cortical knowledge. If one learns something new about one’s own dog, they would not want this knowledge linked to all dogs. The hippocampal system is able to achieve essentially all at once learning without incurring either catastrophic interference or inappropriate, excessively general modification of existing knowledge. It does so by rendering dense connection patterns sparse, thereby substantially eliminating overlapping of representations. The marriage between the cortex and the hippocampus thus enables the brain to achieve the best of both worlds: dense connectivity in the cortex supports overlapping representations and is capable of capturing the statistical structure of experience but at a cost of poor ability to rapidly acquire new declarative knowledge; sparse connectivity in the hippocampus yields minimal overlap of representations and little capacity for capturing the statistical structure of experience but with the enormous benefit of ability to rapidly acquire new declarative knowledge. The orderly interface between cortical and hippocampal systems, originally anticipated by Marr (1971), is now fairly well understood (Rolls, 2016). In turn, the reconciliation of cortical and hippocampal systems in PDP terms provides strong validation of the PDP concept.

Second, we need to briefly inquire as to the constraints that the brain places on where knowledge is stored.

Third, it is worth asking how knowledge is acquired in the first place, what impact this acquisition process has on the scope of the knowledge stored, and how processes of memory consolidation further shape this stored knowledge.

Fourth, the major focus of our discussion of semantic knowledge has been on a system (mammals) that is intrinsically highly hierarchical. However, there are many domains of knowledge that are substantially non-hierarchical. Some discussion of these is essential.

### Episodic Memory Acquisition: Transcending Limitations of Cortical Network Operations

The substantially serial anatomy of the hippocampal system, beginning and ending with the cerebral cortex (cerebral cortex (“what system”/“where system”) → perirhinal/parahippocampal cortex → entorhinal cortex → dentate gyrus → cornu amonis (CA) 3 → CA1 → subiculum → entorhinal cortex → cerebral cortex) reminds us that the hippocampal system stores episodic memories in the form of links between cerebral cortical regions. The approximately 20 million dentate granule cells receive extensive projections from the entorhinal cortex *via* the perforant pathway (Rolls, 2016). This input reflects the extensively overlapping representations supported by the cerebral cortex, almost the entirety of which projects to the entorhinal cortex *via* the perirhinal and parahippocampal cortices. Very rapid competitive processing within the dentate involving inhibitory collateral projections (Gutiérrez, 2003) serves to markedly reduce input overlap and achieve pattern separation. Ongoing neurogenesis in the dentate appears to be essential to the maintenance of this capacity for pattern separation (Spalding et al., 2013; Rolls, 2016). The cerebral cortex, with its extensive autoassociator networks and a high degree of interconnectivity within networks (providing the basis for what are termed *dense* representations), is a highly effective instrument for detecting commonalities between representations. In contrast, processing by the dentate achieves the *sparse* (non-overlapping, orthogonal) representations needed for pattern separation. This sparseness is further enhanced by the very limited but powerful excitatory projections (*via* the mossy fibers) from any given granule cell to a small number of CA3 pyramidal neurons, and by the fact that CA3 pyramidal neurons respond only to the strongest inputs from the dentate. This ingenious system serves a foundational purpose. In the cortex, the overlap between representations is essential to our capacity for building up general (semantic) knowledge from a series of individual experiences, the hierarchical organization of semantic domains, and content addressable memory. However, for learning to be specific to particular semantic exemplars, characteristics of exemplars, or individual experiences, the overlaps between representations supported by cortical knowledge must be minimalized so that what is learned does not apply to entire semantic domains. Furthermore, the overlap must be minimalized so that new knowledge can be acquired without replacing old knowledge, i.e., catastrophic interference (McCloskey and Cohen, 1989; McClelland et al., 1995). The dentate-CA3 system substantially (although not always completely (Norman, 2010) eliminates the overlaps and achieves pattern separation (Brickman et al., 2014; O’Reilly et al., 2014; Rolls, 2016).

The CA3 field is characterized by an extensive recurrent collateral system that spans its length, creating a single autoassociator network with all the properties discussed above (Rolls, 2016). The attractor state into which this ultimately settles represents the point in CA3 activity hyperspace that best reflects the conjunction of the strongest features of the myriad cortical representations engaged at that moment (those that survived the dentate-CA3 gauntlet). These reflect not just the semantic information that is at play in what is to be learned, but also the effects of attention, intention, and the influence of subjective value mediated by input from the orbitofrontal limbic system, as well as the specific and general learning contexts, including time and place (Glenberg, 1979; Glenberg and Lehmann, 1980). CA1 or CA3-CA1 connectivity appears to play a particular role in the encoding of temporal sequences, which are population encoded (Eichenbaum, 2013; Ranganath and Hsieh, 2016). The acquisition of new episodic memories is achieved through very rapid alterations in the synapses of recurrent CA3 collaterals. Entorhinal to CA3 connectivity enables recollection and, because CA3 functions as a single autoassociator network, full memory retrieval can be achieved from a partial cue. In the process of memory consolidation, hippocampally stored episodic memories are gradually transferred to the cerebral cortex to the extent that they share features with knowledge stored in cortical networks (McClelland et al., 1995; Winocur et al., 2010; Kumaran et al., 2016). This gradual *interleaving* of hippocampal input with repeated input from other cortical regions in the course of routine mental processing (and especially during sleep) serves to avoid catastrophic interference (the replacement of old knowledge with new (McClelland et al., 1995). However, it has recently been shown that, if feature sharing between newly learned items and cortically instantiated knowledge structures is extensive, transferral to cortex can occur very rapidly (within 48 h; Tse et al., 2007, 2011) without catastrophic interference (McClelland, 2013).

The gradual transfer of knowledge dependent on hippocampal connectivity to cerebral cortical connectivity, instantiated in the process of interleaved learning, accounts for the phenomenon of temporally graded retrograde amnesia (McClelland et al., 1995). Hippocampal knowledge that cannot be cortically encoded at all remains hippocampally dependent indefinitely and is lost with hippocampal lesions, the most dramatic example being the loss of autobiographical memory with anoxic injury (Vargha-Khadem et al., 1997).

This understanding of hippocampal function reveals how the neurodynamical operations of the heavily interconnected cerebral cortex (intrinsic to which are all of the powerful properties discussed in the foregoing) are transformed into a fundamentally different mathematical form in order to meet several very specific goals while avoiding limitations of and consequences for its operations if such a transformation did not occur. These include (1) sparcification of representations by the dentate-CA3 process so that new episodic memories are highly specific to certain cortical representations and are not mistakenly generalized to large domains, e.g., animals in lieu of my pet cat; (2) capacity for formation of arbitrary associations between objects, times and places by the CA3 autoassociator network; (3) retention of knowledge as long-loop cortico-cortical connectivity until, to the extent that features are shared, it can gradually be integrated into cortical connectivity in the process of memory consolidation while not obliterating old knowledge in the process; (4) the critical facility for full retrieval of a hippocampally dependent memory given only a cue, and (5) the facility for lifetime retention of episodic memories that cannot be integrated into cerebral cortical connectivity.

### The Location of Stored Knowledge: The Connectivity Principle

Knowledge is acquired one experience at a time and patterns of cerebral connectivity determine where this knowledge is stored. This can be illustrated with some examples. The daily business of neurons in auditory association cortices is the processing of acoustic input. In the left hemisphere, to a greater extent than in the right, the daily business of neurons in Broca’s area is to translate input into spoken words. The network of connections between auditory association cortex and Broca’s area, including Wernicke’s area and the supramarginal gyrus, in the course of language learning, acquires knowledge of the orderly relationships between acoustic phonological sequences and spoken articulatory sequences (Plaut et al., 1996; Roth et al., 2006; Nadeau, 2012). Unimodal and polymodal association cortices acquire knowledge of the world and the objects within it through the repeated sensory input that underlies perception. Connectivity between both unimodal and polymodal association cortices and the perisylvian phonological cortex entrains phonological processing to semantic knowledge. Analogous principles of cortical connectivity apply to all components of language function, including syntax and grammatical morphology.

The connectivity principle extends to other regions of the brain. The major inputs to the frontal lobes are sensory (relayed from postcentral association cortices to dorsolateral frontal cortex) and limbic (relayed from limbic structures to the orbitofrontal cortex). The major output of the frontal lobes is to the motor cortex. Thus, the prefrontal cortex is predestined by its connectivity patterns to acquire information that enables the orderly translation of sensory and limbic input into orderly plans for action. The connectivity principle was well understood by Norman Geschwind (1965). The fact that frontal-postcentral connectivity is bidirectional conveys additional capacities for working memory and volitional attention and intention that are essential to the optimization of information processing and to thinking. The connectivity principle constrains the function of auto-associator networks *via* connections between individual networks within neural ensembles (e.g., the different domains of dog knowledge) and the pattern associator networks that translate representations in one domain into representations in another domain, e.g., word meaning into a sequence of phonemes.

The specific nature of the knowledge encoded in the connectivity within any given neural network depends upon; (1) the structure of the knowledge in the domains linked by the connections; and (2) as yet poorly defined hemispheric advantages in processing certain types of data. An example of #1 is provided by networks supporting reading. Orthographic-phonologic connectivity captures the extensive regularities in the relationships between letter strings and phoneme strings. On the other hand, orthographic-semantic-phonologic connectivity involves largely orthogonal knowledge domains and the resilience of knowledge in these domains is almost exclusively dependent on familiarity (the individual counterpart to frequency) and AOA. An example of #2 is provided by word reading and facial recognition. There is evidence that both the fusiform face area and the visual word form area in the right and left inferior temporal lobes, respectively, are involved in both reading and facial recognition (Behrmann and Plaut, 2013), as would be expected given that both processes require analysis of certain visual features held in common. However, as the ability to read develops, thereby instantiating ever more orthographic-phonologic sequence knowledge in the left hemisphere, there is increasing lateralization of face recognition, in many respects a more Gestalt process, to the right hemisphere (Behrmann and Plaut, 2013). However, because reading by the whole word route, in essence, treats words as pictures that elicit corresponding semantic representations, one would predict persistence of some Gestalt processing capacity in the left temporal lobe, hence some capacity for contribution to face recognition; this is borne out by the fact that prosopagnosia tends to be worse with bilateral than with unilateral right inferior temporal lesions. The evolution of hemispheric patterns of superiority in encoding certain types of knowledge may have to do with general hemispheric patterns of white matter connectivity that favor acquisition of sequence knowledge by the left hemisphere (favoring reading by the phonologic route) and acquisition of Gestalt knowledge by the right hemisphere (favoring face recognition; Nadeau, 2010).

### Limitations in the Scope and Depth of Knowledge Acquired Through Experience

We tend to harbor the conceit that our personal knowledge is veridical. I will forego a discussion of the vicissitudes of perception and the impact of attentional processes on the quality of knowledge that was acquired in the first place. Once factual knowledge is acquired by the hippocampal system, it is susceptible to modification by processes of memory consolidation through which hippocampally-dependent facts are gradually encoded in cerebral cortex to the extent that they share features with cortical knowledge (Squire and Zola-Morgan, 1991; Alvarez and Squire, 1994; McClelland et al., 1995; Rolls, 2016). In this process, information on situational context (relation to other stimuli present at the time of acquisition) and general context (time, place, life circumstances, mood, background events of that day, etc.), which were also encoded at the moment of the experience, tends to be lost. Whereas mechanisms of synaptic homeostasis (Tononi and Cirelli, 2014), to the extent that we understand them, appear to work to preserve both knowledge of what is most important and the capacity for further tweaking of neural connectivity to store new knowledge, it is likely that some potentially important knowledge is degraded. This might be most evident to people who have made major career changes, in which case knowledge relevant to a prior career or career phase may be disproportionately lost because it is seldom revisited.

The relatively recent discoveries that memory can be quite rapidly consolidated provided that the new knowledge shares extensive features with existing, cortically instantiated knowledge structures (Tse et al., 2007, 2011; McClelland, 2013), also have implications for what we ultimately know. If cortically compatible episodic memories are rapidly cortically encoded whereas fundamentally new knowledge is subject to the vicissitudes of memory consolidation extending over months to years, it seems likely that our brains will be biased toward learning things that are consistent with what we already know (Tse et al., 2007).

Because knowledge is acquired one experience at a time, what we know is defined by the range of our individual experience and statistical regularities in the stimuli we are exposed to (Plaut and Vande Velde, 2017). This principle might help to account for the diversity of conclusions that different people draw from what is assumed, incorrectly, to be the same knowledge base. It also provides a plausible account for a host of studies on magnitude estimation (Stevens, 1957, 1970). For many types of stimuli in a variety of modalities, humans tend to underestimate the size of high magnitude stimuli (e.g., high pitch, loud sounds, and long lines or, by implication, long edges) and overestimate the size of low magnitude stimuli (e.g., low pitch, quiet sounds, and very short lines; Nadeau, 2014). Because magnitude estimation is always relative to our own life-time experience with a given type of stimulus in particular contexts, stimuli of sizes beyond our experience, high or low, will tend to be under- or overestimated, respectively. In neurodynamical terms, extreme stimuli are so atypical that they fall beyond the outer limits of the attractor basins that correspond to the domains of relevant knowledge and our estimation of them is based upon the extent of the attractor basins that correspond to what we know, or at least know with some measure of confidence.

These principles can also provide a neurally based explanation for the phenomenon of hemispatial neglect that is most dramatic after the right hemisphere strokes (Nadeau, 2014). The realization of a stimulus as a pattern of neural activity does not occur in the absence of attention to it. This was elegantly demonstrated by Moran and Desimone (1985): red light-responsive neurons in the macaque inferior temporal cortex did not respond to red stimuli when the monkey had learned that only responses to green stimuli yielded a reward. Hemispatial neglect usually reflects, at least in part, impairment in attentional systems. In this setting, attractor basins will become shallower (Rolls and Deco, 2015), no less than in semantic dementia, and representations of atypical stimuli, e.g., very long and very short lines, will be lost. On line bisection tasks, patients with hemispatial neglect bisect to the right of the midline, apparently perceiving the attentionally attenuated left side of the line to be exactly as long as the right side of the line. The magnitude estimation literature, and the hypothesis I have proposed to account for it, would lead one to expect that patients with hemispatial neglect would also overestimate small magnitudes in the left hemispace. This is precisely what has been demonstrated in the line-bisection literature in the well-known “cross-over” effect (Tegnér and Levander, 1991; Mennemeier et al., 2005): when the lines to be bisected are sufficiently short, these patients will err by placing the bisection mark to the *left* of midline.

The acquisition of knowledge one experience at a time and the evolution of the corpus of cerebrally instantiated knowledge over the lifetime yield operational characteristics understood through the lens of PDP that provide a logical explanation for the stages of cognitive development first mapped by Piaget (1936) and considerably elaborated since (Munakata et al., 1997; Rogers and McClelland, 2008; Schapiro and McClelland, 2009).

Much knowledge in population encoding neural networks is intrinsically hierarchical. We saw this in our discussion of what happens to semantic networks in semantic dementia. The intrinsically hierarchical nature of knowledge is a direct manifestation of the fact that increasing the specificity of an exemplar requires the addition of features (compare Pekingese with dog), and of the capacity of population encoding networks to capture statistical regularities in their learning experience and instantiate them in neural network connectivity (Nadeau, 2014). This knowledge hierarchy needs no spatial explanation, e.g., that the temporal pole plays an essential role in semantic function (Lambon Ralph et al., 2017). Rather, the particular roles of the temporal pole, e.g., in proper noun knowledge (Miceli et al., 2000), should be sought in the connectivity principle: the particular connectivity of the temporal pole to the amygdala, perirhinal cortex, and orbitofrontal cortex—as well as visual and auditory association cortices. Clearly, the temporal pole is best viewed as a polymodal cortex.

The idea of a special role for the temporal pole in semantics has been inspired by the results of morphologic imaging studies in patients with semantic dementia, bolstered by a computational model that posited an amodal (rather than polymodal) semantic network located in the temporal pole (Rogers et al., 2004). In anatomic studies, the particularly severe atrophy of the temporal pole represents the most readily detectable tip of the iceberg of temporal lobe damage. Anatomic and functional imaging studies actually provide compelling evidence of pathology involving the lateral and inferior temporal cortices through much of their extent (Diehl et al., 2004; Grossman and Ash, 2004; Desgranges et al., 2007), usually sparing the most posterior portions of temporal cortex (Lambon Ralph et al., 1999), which are associated with object gnosis, visual perceptual processes, and perceptual invariance (Hovius et al., 2003; Rogers et al., 2006). This becomes particularly clear when one realizes that the loss of the manner component of verb meaning by patients with semantic dementia (Breedin et al., 1994; Breedin and Martin, 1996; Marshall et al., 1996) likely reflects damage to the human counterpart of area MT located far more posteriorly, at the temporo-parieto-occipital junction, an area that has been implicated in movement perception (Gilmour et al., 1994), action recognition (Kalénine et al., 2010), and action naming (Tranel et al., 2008) and that is engaged by motion verbs in functional imaging studies (Kemmerer et al., 2008). Semantic impairment does occur following left anterior temporal lobe resection but it is not of the severity seen with semantic dementia (Lambon Ralph et al., 2012).

### Non-hierarchical Modes of Knowledge Storage

There are forms of semantic knowledge that reflect associative links between networks rather than hierarchical relationships within networks. This is the case in the relationships between the different component networks of neural ensembles discussed above. Associative relationships between networks also provide the basis for: (1) knowledge underlying abstract words; (2) the relationship between verb knowledge and noun knowledge; (3) contextual knowledge; (4) knowledge of the components of concrete entities; (5) the fact that meaning is often dependent upon circumstance (a knife can be used to cut butter or as a murder weapon) and in the case of homonyms (e.g., the two meanings of “bark”); and (6) the fact that there exist many arbitrary associations borne of experience or metaphor.

The neural representation of abstract words remains a contentious topic. However, a plausible argument can be made that abstract words derive their meaning from contextual associations with concrete and abstract entities. For example, we understand the word “intellectual” in terms of its association with various things like academic institutions, books, esoteric discourse, and smart people, as well as other abstract entities like thinking. Abstract words are related to each other and to concrete words to the extent that they share contextual association (e.g., gamble, casino, poker, chance, luck; Crutch and Warrington, 2005, 2007; Crutch et al., 2009) whereas concrete words are related to each other to the extent that they share semantic features (e.g., yacht, dinghy, canoe, ferry, and barge).

Nouns prime the verbs that they are most often associated with McRae et al. (2005) and verbs prime the nouns that are most often associated with them, either as an agent, object, or indirect object (Ferretti et al., 2001). This priming reflects the associative links that have been formed between the knowledge substrates for multifocal noun representational ensembles and multifocal verb representational ensembles (Figure 2).

Contextual knowledge is commonly probed with such tasks as the Pyramids and Palm Trees Test. Given a triad of a pyramid, a palm tree and a conifer, the two more closely associated entities are readily apparent even though they share no semantic features. Viewed in a different way, pyramids and palm trees are semantic features of a concrete entity called Egypt that, in a way, is an abstraction. Because the relationships here are entirely associative (resembling abstract words in this respect) but involve concrete entities, I will refer to this as *constract* knowledge. Much of what we know about locations and times may be viewed as constract knowledge and is highly idiosyncratic. For example, the most prominent components of my personal knowledge of Los Angeles consist of one of my daughters, a dear friend in Encino, Norton Simon Museum, Rodin, Nat’n Al Delicatessen, the 405, Huntington Gardens, cacti, a working meeting at the University of Southern California, and many movies. People’s memories of the assassination of John F. Kennedy and 9/11 are substantially comprised of what they were doing at the time of these events. Because such place- and time-specific memories are so idiosyncratic, they are likely to remain substantially hippocampally dependent indefinitely.

The components of concrete entities generally do not share features with the entities themselves. For example, dogs are composed of visceral organs whose functions and cellular processes share no features with dogs. The knowledge linking dogs with hearts, lungs, kidneys, and brain is associative in nature, not hierarchical. Thus, this knowledge is similar in nature to that underlying constract words. However, by and large, it is not idiosyncratic. Further, while this type of knowledge does not represent regularities that have emerged in a population-encoding network, it may represent a domain of knowledge that is deeply coded in neural connectivity by virtue of being shared by so many entities (all animals in this example).

The fact that meaning is often dependent on circumstance reflects another type of associative knowledge (tree bark vs. dog bark; knife on a butter platter vs. knife in “Psycho”).

Some associations are borne of juxtapositions in experience or in metaphor. “Cat” and “dog” are taxonomically distinct but are commonly collocated in a home and maybe metaphorically related, e.g., “fight like cats and dogs.” So too “hot and cold” and “black and white.”

Associational relationships that provide the basis for components of concrete entities, abstract concepts, noun-verb interdependence, and constract words, as well as meanings that are circumstance-specific or reflect frequent juxtaposition or metaphor, are presumably supported by long white matter pathways that link relevant portions of the brain. Because the knowledge of these relationships does not reflect emergent statistical regularities, it must depend primarily on frequency and AOA effects on neural connectivity.

## PDP and the Six-Layered Cerebral Cortex

The evidence of the power conferred by PDP in understanding cortical function enables us to ask some questions about a statement made early in this manuscript: “these simple mathematics obviously do not do justice to all the subtleties of actual neural processing.” It is customary to think of the cerebral cortex as a vast assemblage of 6-layer microprocessors. Is this thinking correct and, if not, to what extent has it inhibited our thinking about the mechanisms of cortical function? Could the cortex be better viewed as fundamentally two 3-layer processors, one centered on layers 2/3, largely responsible for cortico-cortical computation, and the other on layers 5/6, largely responsible for cortical output processes? Rolls has suggested a variation on this theme (Rolls, 2016). There is evidence in mice that the cells of layers 2, 3, and 4 have a different neuroglial origin than the cells of layers 5 and 6 and in birds, the two groups of cells are physically separated (Karten, 2015). The principal operational layer at any one moment could be determined by the noradrenergic system (Devilbiss and Waterhouse, 2000; Devilbiss and Berridge, 2008). Could the layering be, to some extent, merely an adaptive way of efficiently providing vast input to the dendritic arborizations of pyramidal cells in the two levels, as observed with Purkinje cells in the cerebellum? Are the inputs from one layer to another, e.g., from layer 4 of one region to layer 3 of another, data-transformative, as with the entorhinal cortex-dentate nucleus-CA3 pathway, or are they merely the means of linking different auto-associator and pattern-associator networks? In short, are PDP models really as simplistic as they are often thought to be?

## Domains of Specialized Knowledge and Processing

In this section I briefly review a number of domains of cortical function for which investigations predicated upon the concept of population encoding networks have enabled deeper insights into neural mechanisms underlying higher neural functions.

### Language

Language represents our largest window into human cerebral function and it has been the most studied of all higher neural functions. Language function is based upon a number of domains of knowledge, including semantic, phonological sequence, morphological sequence, acoustic-semantic, orthographic-phonologic, orthographic semantic, semantic-phonologic and semantic-morphologic (lexical knowledge: the means by which we translate meaning into articulatory sequences), and acquired knowledge of language-specific habits of ordering and modifying concept representations, the basis for syntax (Nadeau, 2012). We now have a fairly granular understanding of these domains of knowledge supporting language function (McClelland et al., 2014; Seidenberg and Plaut, 2014; Joanisse and McClelland, 2015) and the ways that they interact in a substantial number of languages, particularly as revealed in studies of aphasia (English, Dutch, Italian, German, Spanish, Catalan, Serbo-Croatian, Hungarian, Turkish, Chinese, and Swahili, among others; Nadeau, 2012, 2019; Rogers and McClelland, 2014). The principles implicit in population encoding networks also provide an orderly account for language deficits seen in bilinguals and polyglots with aphasia (Nadeau, 2019). In all of these languages, despite their enormous differences, the attributes of aphasia are readily explained in terms of the unique grammatical structure of the language affected, instantiated in population encoding networks, and the effects of regional damage to the brain mitigated by graceful degradation. PDP principles have also been of value in devising new treatments for aphasia following stroke (Plaut, 1996; Kendall et al., 2008; Edmonds et al., 2009; Kendall et al., 2015; Nadeau, 2015; Nadeau, 2019).

There is evidence that the way we learn and refine our own individual ways of speaking a language, e.g., English, across our lives, reflects not just the evolving chaotic order in our own brains as we acquire new language knowledge from experience, but the language itself, which reflects the chaotic order that evolved among populations of English speakers across the centuries, driven by the various external linguistic forces that buffeted the English language during this time (Hare and Elman, 1992). None of this needs to be logical in any conventional sense, although, as Hare and Elman have shown, the underlying dynamics can be understood logically in PDP terms. Analogous processes may occur in other domains, e.g., in the evolution from homo sapiens perceptualis of Neolithic times, for whom acute perception of the subtlest features of the environment was of survival advantage, to homo sapiens conceptualis of modern times, for whom a conceptual understanding of the world we live in and the vast store of semantic knowledge underlying it, is more important.

### Attention

Computational models employing population encoding have been developed that emulate volitional attention in both the “what” and “where” visual pathways as reflected in single-unit studies in monkeys and functional magnetic resonance imaging (fMRI) studies in humans (Corchs and Deco, 2002; more about the “what” and “where” pathways below in the “Conclusion” section). Simpler computational models have successfully simulated processes involved in reactive attention driven by stimulus salience, familiarity, or context (Spratling and Johnson, 2004). A population encoding neurodynamical model has been developed that accurately simulates the neurophysiological behavior of V2 and V4 neurons and quantitatively replicates the interaction between volitional attentional effects and stimulus salience effects that have been observed in neurophysiological studies in nonhuman primates (Deco and Rolls, 2005).

Population encoding computational models have also been developed that provide a logical, mechanistic and granular account for the effect of focal lesions on attentional systems, producing phenomena such as hemispatial neglect (Mozer, 2002; Rolls and Deco, 2002; Monaghan and Shillcock, 2004) and neglect dyslexia (Moser and Behrmann, 1990).

### Emotional Function

Simulations involving population encoding models have explored fear conditioning (Armony et al., 1997); discrimination of emotional faces (Armony et al., 1997); inference of facial emotional expression from neutral facial features as a function of the gender and race of faces (Zebrowitz et al., 2010); and the enhancement of the Stroop interference effect by emotion words in the context of generalized anxiety disorder, post-traumatic stress disorder, and phobias (Mathews and Harley, 1996). The interaction of anxiety level and threat in influencing visual attentional bias has been explored in a non-population encoding connectionist model (Frewen et al., 2008).

### Executive Function

The Stroop interference effect (processing costs associated with responding “red” when “blue” is written in red ink) has received particular attention. Though beguilingly simple, this test pits the most fundamental of brain processes against each other: volitional attention and volitional intention (voluntary direction of attention to a particular stimulus or stimulus attribute and voluntary formulation of an action plan) against reactive attention and reactive intention (attention to a particular stimulus because of its salience and automatic formulation and execution of the implicit plan)—thinking slow against thinking fast. This dialectic has often been posed as controlled vs. automatic processing. PDP simulations have been particularly informative about what drives stimulus salience (which drives reactive attention). Early studies suggested that mere greater experience (more training reading words than colors) sufficed to emulate human behavior (Cohen et al., 1990, 1992, 1998). However, it subsequently became evident that salience might also be influenced by the nature of the processing, e.g., that reading could occur by a direct orthographic-articulatory route that did not require the generation of semantic representations, whereas color naming has to engage potentially competing semantic representations (Kanne et al., 1998). Other models have attempted to account for intentional components of Stroop performance, most notably the observation that response latency is more prolonged after a switch from color naming to word naming than it is after a switch from word naming to color naming (Gilbert and Shallice, 2002). This phenomenon may reflect the varied nature of persistent distributed response representations (a working memory).

A population encoding model has also been successfully employed to account for performance on the traveling salesperson problem by normal human participants and participants with executive dysfunction (Cutini et al., 2008). This problem requires the selection of the shortest travel route from an initial to a final “city” within a multi-city spatial array.

### Motor Planning

Studies of motor planning and execution have included investigation of the ability to acquire knowledge required to assume specific body postures through imitation and the capacity to generalize from this knowledge to untrained postures and untrained sources of visual input (Chaminade et al., 2008); investigation of action sequence knowledge and the breakdown of sequential performance reflected in action slips in normal people and the action disorganization syndrome in individuals with brain damage (Botvinick and Plaut, 2004); investigation of action selection and object naming from orthographic or visual object input in normal and brain-damaged individuals (e.g., with optic aphasia or visual apraxia; Yoon et al., 2002); and view-independent grip aperture computation (Prevete et al., 2008).

### Visual Processing

Studies of visual function have addressed prosopagnosia and general neural mechanisms underlying differences in overt and covert recognition performance (Farah et al., 1993); facial recognition (Luckman et al., 1995); visual agnosia (Barbeau and Giusiano, 2003); and the interaction between the dorsal “where” visual system and the ventral “what” visual system and the basis for simultanagnosia and illusory conjunctions (erroneous combinations of features of multiple stimuli; Henderson and McClelland, 2011).

### Other

Population encoding model simulations have been employed to study other topics, faithfully recapitulating human behavior and providing new insights into fundamental parameters of that behavior, including olfaction (Haberly, 2001) and creative problem solving (Hélie and Sun, 2010).

There may be additional, so far untapped and perhaps unexpected domains in which PDP approaches can relate observed behavior to neural mechanisms. One example of particularly broad significance is human decision making in the face of uncertainty, a topic at the intersection of psychology, economics, and neuroeconomics. People can, of course, volitionally behave like coldly rational economists and calculate the expected value as the actual value of an outcome multiplied by the probability of its achievement (a volitional intentional process). However, it seems that, in general, they actually arrive at the expected value by relying on their reactive “sense” of probabilities, which is based upon knowledge acquired through experience, however inadequate that might be. This sense follows the familiar pattern of magnitude estimation with the overweighting of low probabilities and underweighting of high probabilities (Kahneman, 2011), which, as discussed above, can be related to the topography of attractor basins (Nadeau, 2014). The consequences are very different for behavior in the linked loss and gain attractor basins (basins corresponding to negative and positive emotional states), loss aversion being far more motivating than gain acquisitiveness. In addition, the attractor basins are shaped by one’s particular circumstances at decision time, the nature and value/disvalue of the outcomes, and the ways in which they are defined (Kahneman, 2011), as with attractor basins in general (Nadeau, 2014). In general, faced with a low probability of a loss (e.g., a 5% chance of losing $10,000), people are risk-averse, inclined to settle for a certain loss considerably greater than the expected negative value (0.05 × 10,000 = 500)—hence insurance. On the other hand, faced with a low probability of gain, people are inclined to be risk-seeking, gambling amounts greater than the expected value (e.g., betting $5 for a one in a million chance to win $1 million)—hence lotteries. Faced with a high probability (or certainty) of a loss, people are nevertheless risking seeking, being willing to gamble excessive amounts if there is a chance of avoiding a certain negative settlement—hence the behavior of the desperate gambler. Faced with a high probability of gain, people are risk-averse, likely to settle for an amount considerably less than the expected value rather than endure a small risk of losing it all (Kahneman, 2011). These examples inspire confidence that even the most complex of human behaviors can be explained in neurodynamical terms.

## Conclusion

### Overview

We have seen that PDP approaches are able to account, without contrivance or algorithmic devices, for an extraordinary variety of well-studied cognitive and behavioral phenomena in normal and brain-injured individuals and to contribute usefully to the scientific dialogue in many domains. It is often worthwhile to consider seemingly incomprehensible cognitive phenomena in PDP terms; even without the use of computer simulations, “PDP thinking” can often render such problems tractable. Population encoding PDP models emulate neural network structure and thus have neural plausibility. In the course of learning, they are able to capture the effects of frequency, age of acquisition, and statistical regularities of experience demonstrated by the brain; herein lies their greatest power. Their most important intrinsic weakness, the inability to rapidly acquire new declarative knowledge as episodic memories, is handily addressed through the unique structure of the hippocampal system. Because of the property of graceful degradation that is intrinsic to PDP models and apparently to the brain, it is possible to “reverse engineer” the normal brain through analysis of the performance of individuals with brain damage.

### The Long View: Way-Stations, Multiservice Function, and Computational Mechanisms

PDP models were originally developed with the goal of understanding how knowledge is represented in the brain and how representations in one domain can be translated into representations in another domain. The employment of simple nonlinear mathematics provided the basis for settling of network activity into attractor states within attractor basins in an energy landscape supported by attractor networks. Prior to the development of PDP, patterns of aberrant behavior associated with brain lesions could only be understood in terms of the destruction of essential neural substrates and disconnection between domains of knowledge as revealed in structural imaging studies. The exact reasons for behavioral phenomena resulting from brain damage, whether it be the destruction of knowledge domains or connections between domains, remained a matter of speculation. Our understanding of PDP has now gone a long way to enabling us to understand the how and why of the degradations of cognitive function observed with brain damage.

However, the science of PDP invites us to extend the fundamental principles much further. Both cognitive neuroscientists and basic neuroscientists tend to study particular regions or structures within the brain in isolation, drawing inferences from the stimuli and the environmental contexts that appear to engage these regions or structures and the resultant behaviors. The science of PDP, however, leads us to understand particular regions or structures as way-stations in multiservice cognitive processing and as components of computational structures. Some examples will help to convey these ideas.

Mishkin et al. (1983) articulated the concept of two visual systems, a ventral “what” system and a dorsal “where” system. The “what” system, supported by the inferior temporal cortex, is fundamentally a single object recognition system. It supports the processing of the detailed color, form, and textural information that instantiates our perception of objects as objects and our visual semantic knowledge of objects. The “where” system supports our perception of the location of objects in egocentric space. The what/where dichotomy was actually presaged by the discovery by Lissauer in 1890 (Lissauer, 1988) of agnosia following ventral occipitotemporal lesions and the discovery by Balint (1909) of optic apraxia, optic ataxia, and simultanagnosia following dorsal occipitoparietal lesions, Nevertheless, the Mishkin et al. (1983) explication was a tour de force and continues to provide the fundament of our understanding of visual processing by the brain.

In 1992, Goodale and Milner (Goodale and Milner, 1992), in a superb analysis still well-worth reading, challenged the characterization of the dorsal system as “where” and suggested a reformulation as “what” and “how” systems, citing, in particular, the influence of occipitoparietal input to frontal systems on grip aperture and alignment in the course of task performance (see also Milner and Goodale, 2008). A great deal of research has followed, refining this idea (Freud et al., 2016). Unfortunately, the “how” characterization seems somewhat inapt. The occipitoparietal cortex enables translation from retinotopic coordinates to spatiotopic coordinates (Wurtz, 2008). It supports our knowledge of the location of objects in space (Brodt et al., 2016). By virtue of binocular vision, it provides the capacity for depth perception. By virtue of connectivity between the occipitoparietal cortex and the inferotemporal cortex (Milner, 2017), we have the illusion of photographic perception despite the fact that the “what” system is a single object processing system. Parieto-occipital lesions, presumably by disrupting this connectivity, yield simultanagnosia, which may reflect a disorder of parietally mediated attention (Dalrymple et al., 2013) or a disorder of occipitoparietal-inferior temporal interaction (Spratling and Johnson, 2004; Coslett and Lie, 2008; Rolls, 2016). The dorsal system supports reactive attention drawn by the occurrence of salient stimuli at particular locations in space and volitional attention to particular regions of space driven by frontal input. In short, the occipitoparietal cortex is a *multiservice* structure subserving multiple “where” functions. However, by dint of the extensive projections from postcentral cortices to frontal cortex first discovered by Chavis and Pandya (1976), post-central structures are well suited to influencing the formulation and execution of frontally based plans for action. Objects in egocentric space subtend a visual arc, defined by edges and textural contrasts. These provide the basis for the influence of the occipitoparietal cortex on grip aperture and orientation (Goodale and Milner, 1992). In this particular function, the occipitoparietal cortex serves as a *way-station* (an interposed hidden unit pool) to frontal motor function.

While there is general agreement as to the function of the inferotemporal “what” system, it too serves as a way-station. The perception of a dog elicits a detailed population encoded representation of a dog. However, it also elicits representations corresponding to dog knowledge in general. It may engage the hippocampal system (“I think this is the dog I saw over at Johnson’s house the other day”). It is likely to engage the limbic system, either positively or negatively. If the dog appears friendly, expectant and is wagging its tail, it elicits warmth and happiness and it may elicit approach behavior in frontal systems pursuant to petting. If the dog is rigid, trembling, teeth bared, and snarling, it may elicit a feeling of terror and the engagement of frontally based plans to flee. Thus, the “what” system also serves as a way-station to limbic and frontal processing.

For some 50 years, the basal ganglia have been intensively studied, in good part because of the consequences of their dysfunction evident in Parkinson’s disease. The overwhelming preponderance of the evidence is that the sensorimotor basal ganglia serve a motor function, as yet not well understood. However, recent studies from PDP and evolutionary perspectives suggest that the basal ganglia have nothing to do with motor function *per se* (Fiore et al., 2015; Nadeau, 2020). Rather, in creatures ranging from arthropods (yes, bugs have basal ganglia) to primates, the basal ganglia system (cortex, striatum, globus pallidus interna and externa, subthalamic nucleus, thalamus, cortex) serves as a computational device for dimensionality reduction. The sensorimotor basal ganglia take a vast multidimensional polymodal array of sensory input and translate it, through settling into particular attractor trenches, into a limited number of optimal, mutually compatible movements selected from a limited motor repertoire. In simple animals, e.g., lampreys, the sensory input is as vast and complex as in humans but the motor repertoire is extremely limited (Fiore et al., 2015). In humans, with their vast behavioral repertoire and their mechanisms for selectively engaging sensory cortices (attention and working memory), there is reason to question the utility of the sensorimotor basal ganglia; this argument receives support from the results of pallidotomy used to treat Parkinson’s disease, which, in perfectly treated patients, may yield apparently normal function (Nadeau, 2020). Most importantly, any one structure in the basal ganglia system, e.g., the striatum, serves only as a way-station.

Even relatively simple PDP networks perform computational functions. Attractor networks enable settling into attractor basins and ultimately attractor states depending on the configuration of input. Pattern associator networks provide an orderly translation of representations in one knowledge domain, e.g., semantics, into another knowledge domain, e.g., phonology. Other systems perform more complex computational functions. As already discussed, the hippocampal system subserves a complex computational function that makes possible the encoding of episodic and long-term declarative memory and the basal ganglia system subserve a computational process of dimensionality reduction that provides the major basis, at least in lower animals, for reactive intention. The occipitoparietal cortex appears to support a computational process that enables the detection of edges, changes in texture, and changes in internal configuration (Zachariou et al., 2015, 2017; Freud et al., 2016), much like the mathematical function of a Gabor filter^1^.

### Possible Reasons for Lack of Penetration of PDP Concepts Into Cognitive Psychology, Cognitive Neuropsychology, and Neuropsychology

A number of reasons can be identified. Some are related to PDP science itself. Population encoding models are still commonly viewed as just one more heuristic approach and a difficult one at that because of their mathematical instantiation, their development in computer simulations, and their sometimes opaque or counterintuitive characteristics. As I have sought to show in this article, PDP is anything but a heuristic approach. Even models incorporating the very simple mathematics discussed in the first section provide a unitary explanation for a vast array of well-established properties of brain systems. Against expectation, PDP constitutes something of an Occam’s razor. Furthermore, PDP concepts can be applied in the absence of mathematical skills or computer simulations. PDP has often been rejected out of hand on the basis of limitations in the design of specific models (often intended), the flaws of specific models, or the weakness of the scientific data that were employed to test particular models. Because PDP simulations generate voluminous data and very specific predictions for behavior, they are particularly susceptible to detailed criticism, in contrast to the often rather general and underspecified arguments that may be leveled at PDP in general. It is not always recognized that any specific PDP model constitutes a very explicit hypothesis.

Advances in this field have been hindered by the still ubiquitous problems of communication across scientific disciplines. Most notably, PDP scientists have made only a modest number of forays into the science of broken brains (Farah and McClelland, 1991; Plaut and Shallice, 1991, 1993a,b; Farah et al., 1993; Plaut, 1996; Plaut et al., 1996; Rogers and McClelland, 2004; Cutini et al., 2008; Henderson and McClelland, 2011; Rogers et al., 2015). The result is that PDP concepts are largely foreign to the fields of neuropsychology and cognitive neuropsychology (the latter arguably the single greatest contributor to our current understanding of how the brain supports cognitive function), as well as much of cognitive psychology. There have been many PDP simulations seeking to account for behavior, usually in normal people, many quite successful, but these have been largely proof of concept studies that have not ascertained the value of the powerful and fundamentally statistical intrinsic properties of population encoded representations—the central focus of this article.

Some domains of cognitive neuroscience have been dominated by competing models, the most conspicuous example being Chomskian linguistics in the case of language (despite its utter lack of neural plausibility).

Arguably the most serious impediment to acceptance of PDP has been the dominance of cognitive neuroscience by functional imaging over the past 25 years. The appeal of “seeing the brain think,” the notion that functional imaging results are necessary to validate conclusions borne of careful, hypothesis-driven psychological studies, the rapid development of very sophisticated image acquisition and processing methodologies, the ubiquity of magnetic resonance imaging (MRI) devices, and the dominance of study sections by “imagers” have all played a role. However, functional imaging is fraught with serious problems. First and most fundamentally, whereas, as we have seen, representations in the brain are highly distributed as patterns of activity involving large areas of the brain and involving billions of neurons, the statistical parametric mapping (SPM) algorithm that underlies fMRI processing is hyper-localizing in that, by design, it seeks to identify localized regions of brain associated with particular functions. This problem is compounded by the limited sensitivity of the method. The net result is that what is actually imaged represents the tip of an iceberg of synaptic activity (incentivizing the concept of functional“nodes”). Entire cerebral functions are often linked to these tips—conclusions markedly at odds with the understanding that has emerged from PDP that the brain is a mass of hidden units engaged in processes and computations. Second, fMRI signal (e.g., blood oxygen level-dependent, BOLD) is predominantly generated by neural synaptic activity, which is the major source of neural energy consumption (Schwartz et al., 1979; Mata et al., 1980). This means that, at least in the cortex, areas of increased signal indicate areas of increased afferent input rather than increased neuronal activity *per se*. This renders the interpretation of these imaging findings more difficult.

fMRI also suffers from serious problems of experimental control, for at least five reasons. First, while the average participant no doubt strives to correctly perform the assigned task, the processes occurring in their brain remain a matter of some speculation and may vary from participant to participant, session to session, and within a session. Second, regions of activation correspond to regions of maximal synaptic activity; what exact role these regions might play in multi-stage, multifocal cerebral processes is not defined. Third, it is seldom possible to distinguish regions of synaptic activity that are essential to a given function from those that are incidental. Fourth, despite the enormous technological sophistication of current functional imaging methods, we still routinely see areas of “activation” located within the cerebrospinal fluid of the ventricles or within white matter, which has no synapses. Fifth, the statistics of SPM are largely the statistics of voxels, not human populations. For this reason, it is rarely possible to determine to what extent the findings of a functional imaging study are idiosyncratic to the group of participants studied.

The results of resting-state fMRI studies have proven remarkably reproducible, even as this approach implicitly gives up on experimental control entirely. Resting-state studies have provided the major setting for studies of functional connectivity. However, they ignore the fact that functional connectivity is state-specific (e.g., the competition of movement representations and motion verb representations for motor cortex discussed above). Furthermore, functional connectivity might be substantially defined by processes, such as electroencephalographic rhythms, that provide the basis for correlations between synaptic activity in different regions of the brain but have only an indirect relationship to discrete functions.

Finally, functional imaging has generally sought to answer “where” questions, even as most “where” questions can be answered on the basis of the connectivity principle (bolstered by results of diffusion tensor tractographic studies) and have been addressed by lesion studies. Prosopagnosia was differentially linked to lesions of the right posterior inferior temporal region by Bodamer (1947) long before the “fusiform face area” was described. Alexia was differentially linked to lesions of the left posterior inferior temporal region by Dejerine (1892) long before the “visual word form area” was “discovered.” However, “where?” studies may be of value if hypothesis-driven (see examples in the next paragraph).

fMRI is extremely complex and the methodological challenges almost certainly will never be fully addressed. Nevertheless, there have been many functional imaging studies that have made important contributions to cognitive neuroscience. The major distinguishing feature of these studies is that they have been hypothesis-driven (see also Coltheart, 2006, 2013; Tressoldi et al., 2012). This has meant often extraordinary efforts to achieve experimental control together with analyses focused on specific regions of interest. fMRI studies that advance science also tend to ask “what,” “how,” “why,” and “in what way” questions rather than “where” questions. What we know of the answers to such questions derives predominantly from “low tech” cognitive neuropsychological studies. Because of the principle of graceful degradation, related to the distribution of knowledge within networks and across the networks of a neural ensemble, the unique contributions of particular brain regions, as well as regularity, frequency, and age of acquisition effects are often unmasked. For this reason, precise, tightly controlled, hypothesis-driven cognitive neuropsychological studies carried out over extended periods of time and in many participants can be particularly revealing about the how and the why, particularly when viewed through the lens of population encoding principles. fMRI can contribute to this what/how/why/in-what-way query but only through carefully designed hypothesis-driven studies that achieve sufficient experimental control. There are many such studies but I will cite four in particular by way of example. Work by Kemmerer et al. (2008) has contributed to our understanding of the different components of verb representations. Studies have confirmed that the implementational component of verb representations is somatotopic (Kemmerer et al., 2008; Raposo et al., 2009; Kemmerer and Gonzalez-Castillo, 2010). Wu et al. (2008) demonstrated that cerebral instantiation of manner and path (key components of verb representations) was consistent with our understanding of the representation of intrinsic movement in the human homolog of area MT (the occipital-temporal-parietal junction) and the representation of movement in egocentric space in parietal cortex. Two studies have strongly implicated the supragenual anterior cingulate region in motor plan gating (Iadarola et al., 1998; Peyron et al., 1999). While these studies likely do not meet Coltheart’s standards (Coltheart, 2006, 2013; Tressoldi et al., 2012), they have nevertheless advanced our understanding of the cerebral underpinnings of cognitive function.

In closing, I note that, although study sections, editors and reviewers consistently demand motivating hypotheses, what often passes for a hypothesis is merely a prediction, hardly more than wishful thinking. A hypothesis is useful only to the extent that it is mechanistically based. Cognitive neuropsychology has flourished using hypotheses based upon information processing models, their limitations notwithstanding. Innumerable articles on language function that have been motivated by Chomskian theory have yielded very important insights, despite the lack of neural plausibility of the model, because *a priori* hypotheses led to tight experimental control. PDP provides a powerful mechanistic basis for hypothesis generation.

## Author Contributions

The entire manuscript was conceived and written by SN.

## Conflict of Interest

The author declares that the research was conducted in the absence of any commercial or financial relationships that could be construed as a potential conflict of interest.

## Footnotes

^1^https://cvtuts.wordpress.com/2014/04/27/gabor-filters-a-practical-overview/

## References

[B1] Alvarez P. Squire L. R. (1994). Memory consolidation and the medial temporal lobe: a simple network model. Proc. Natl. Acad. Sci. U S A 91, 7041–7045. 10.1073/pnas.91.15.7041 8041742 PMC44334

[B2] Armony J. L. Servan-Schreiber D. Cohen J. D. LeDoux J. E. (1997). Computational modeling of emotion: explorations through the anatomy and physiology of fear conditioning. Trends Cogn. Sci. 1, 28–34. 10.1016/s1364-6613(97)01007-3 21223850

[B3] Balint R. (1909). Seelenlahmung des “Schauens”, optische Ataxie, raumliche Störung der Aufmerksamkeit. Monatsschr. Psychiatr. Neurol. 25, 57–71.

[B4] Barbeau E. Giusiano B. (2003). Category-specific visual agnosia: lesion to semantic memory versus extra-lesional variables in a case study and a connectionist model. Brain Cogn. 53, 433–440. 10.1016/s0278-2626(03)00215-x 14642293

[B5] Behrmann M. Plaut D. C. (2013). Distributed circuits, not circumscribed centers, mediate visual recognition. Trends Cogn. Sci. 17, 210–219. 10.1016/j.tics.2013.03.007 23608364

[B6] Bodamer J. (1947). Prosopagnosie. Arch. Psychiatr. Nervenkr. 179, 6–54. 10.1007/BF00352849

[B7] Botvinick M. Plaut D. C. (2004). Doing without schema hierarchies: a recurrent connectionist approach to normal and impaired routine sequential action. Psychol. Rev. 111, 395–429. 10.1037/0033-295x.111.2.395 15065915

[B8] Boulenger V. Shtyrov Y. Pulvermüller F. (2012). When do you grasp the idea? MEG evidence of instantaneous idio understanding. NeuroImage 59, 3502–3513. 10.1016/j.neuroimage.2011.11.011 22100772

[B10] Breedin S. D. Martin R. C. (1996). Patterns of verb impairment in aphasia: an analysis of four cases. Cogn. Neuropsychol. 13, 51–92. 10.1080/026432996382060 28532314

[B9] Breedin S. Saffran E. M. Coslett H. B. (1994). Reversal of the concreteness effect in a patient with semantic dementia. Cogn. Neuropsychol. 11, 617–660. 10.1080/02643299408251987

[B11] Brickman A. M. Khan U. A. Provenzano F. A. Yeung L.-K. Suzuki W. Schroeter H.. (2014). Enhancing dentate gyrus function with dietary flavanols improves cognition in older adults. Nat. Neurosci. 12, 1798–1803. 10.1038/nn.3850 25344629 PMC4940121

[B12] Brodt S. Pöhlchen D. Flanagin V. L. Glasauer S. Gais S. Schönhauer M. (2016). Rapid and independent memory formation in the parietal cortex. Proc. Natl. Acad. Sci. U S A 113, 13251–13256. 10.1073/pnas.1605719113 27803331 PMC5135314

[B13] Carreiras M. Armstrong B. C. Perea M. Frost R. (2014). The what, when, where, and how of visual word recognition. Trends Cogn. Sci. 18, 90–98. 10.1016/j.tics.2013.11.005 24373885

[B14] Carreiras M. Mohahan P. J. Lizarazu M. Duñabeitia J. A. Molinaro N. (2015). Numbers are not like words: different pathways for literacy and numeracy. NeuroImage 118, 79–89. 10.1016/j.neuroimage.2015.06.021 26067344

[B15] Chaminade T. Oztop E. Cheng G. Kawato M. (2008). From self-observation to imitation: visuomotor association on a robotic hand. Brain Res. Bull. 75, 775–784. 10.1016/j.brainresbull.2008.01.016 18394524

[B16] Chavis D. A. Pandya D. N. (1976). Further observations on corticofrontal connections in the rhesus monkey. Brain Res. 117, 369–386. 10.1016/0006-8993(76)90089-5 825194

[B17] Churchland P. S. Sejnowski T. J. (1992). The Computational Brain. Cambridge, MA: MIT Press.

[B19] Cohen J. D. Dunbar K. McClelland J. L. (1990). On the control of automaticity processes: a parallel distributed processing account of the Stroop effect. Psychol. Rev. 97, 332–361. 10.1037/0033-295x.97.3.332 2200075

[B18] Cohen J. D. Servan-Schreiber D. McClelland J. L. (1992). A parallel distributed processing approach to automaticity. Am. J. Psychol. 105, 239–269. 10.2307/1423029 1621882

[B20] Cohen J. D. Usher M. McClelland J. L. (1998). A PDP approach to set size effects within the Stroop task: reply to Kanne, Balota, Spieler and Faust (1998). Psychol. Rev. 105, 188–194. 10.1037/0033-295x.105.1.188 9450376

[B21] Coltheart M. (2006). What has functional imaging told us about the mind (so far)? Cortex 42, 323–331. 10.1016/s0010-9452(08)70358-7 16771037

[B22] Coltheart M. (2013). How can functional neuroimaging inform cognitive theories? Perspect. Psychol. Sci. 8, 98–103. 10.1177/1745691612469208 26172256

[B23] Corchs S. Deco G. (2002). Large-scale model for visual attention: integration of experimental single-cell and fMRI data. Cereb. Cortex 12, 339–348. 10.1093/cercor/12.4.339 11884349

[B24] Coslett H. B. Lie C. (2008). Simultanagnosia: when a rose is not red. J. Cogn. Neurosci. 20, 36–48. 10.1162/jocn.2008.20002 17919075

[B27] Crutch S. J. Connell S. Warrington E. K. (2009). The different representational frameworks underpinning abstract and concrete knowledge: evidence from odd-one-out judgments. Q. J. Exp. Psychol. 62, 1377–1390. 10.1080/17470210802483834 19096991

[B25] Crutch S. J. Warrington E. K. (2005). Abstract and concrete concepts have structurally different representational frameworks. Brain 128, 615–627. 10.1093/brain/awh349 15548554

[B26] Crutch S. J. Warrington E. K. (2007). Semantic priming in deep-phonological dyslexia: contrasting effects of association and similarity upon abstract and concrete word reading. Cogn. Neuropsychol. 24, 583–602. 10.1080/02643290701577351 18416510

[B28] Cutini S. Di Ferdinando A. Basso D. Bisiacchi P. S. Zorzi M. (2008). Visuospatial planning in the travelling salesperson problem: a connectionist account of normal and impaired performance. Cogn. Neuropsychol. 25, 194–217. 10.1080/02643290701606408 18568813

[B29] Dalrymple K. A. Barton J. J. S. Kingston A. (2013). A world unglued: simultanagnosia as a spatial restriction of attention. Front. Hum. Neurosci. 7:145. 10.3389/fnhum.2013.00145 23616758 PMC3627977

[B30] Deco G. Rolls E. T. (2005). Neurodynamics of biased competition and cooperation for attention: a model with spiking neurons. J. Neurophysiol. 94, 295–313. 10.1152/jn.01095.2004 15703227

[B31] Dejerine J. (1892). Contribution à l’étude anatomopathologique et clinique des différent variétés de cécité verbale. C.R. Séances Soc. Biol. 4, 61–90.

[B32] Desgranges B. Matuszewski V. Piolino P. Chétat G. Mézenge F. Langeau B.. (2007). Anatomical and functional alterations in semantic dementia: a voxel-based MRI and PET study. Neurobiol. Aging 28, 1904–1913. 10.1016/j.neurobiolaging.2006.08.006 16979268

[B33] Desimone R. Duncan J. (1995). Neural mechanisms of selective visual attention. Annu. Rev. Neurosci. 18, 193–222. 10.1146/annurev.ne.18.030195.001205 7605061

[B35] Devilbiss D. M. Berridge C. W. (2008). Cognition-enhancing doses of methylphenidate preferentially increase prefrontal cortex neuronal responsiveness. Biol. Psychiatry 64, 626–635. 10.1016/j.biopsych.2008.04.037 18585681 PMC2603602

[B34] Devilbiss D. M. Waterhouse B. D. (2000). Norepinephrine exhibits two distinct profiles of action on sensory cortical neuron responses to excitatory synaptic stimuli. Synapse 37, 273–282. 10.1002/1098-2396(20000915)37:4<273::aid-syn4>3.0.co;2-# 10891864

[B36] Diehl J. Grimmer T. Drzezga A. Riemenschneider M. Förstl H. Kurz A. (2004). Cerebral metabolic patterns at early stages of frontotemporal dementia and semantic dementia. A PET study. Neurobiol. Aging 25, 1051–1056. 10.1016/j.neurobiolaging.2003.10.007 15212830

[B37] Edmonds L. A. Nadeau S. E. Kiran S. (2009). Effect of verb network strengthening treatment (VNeST) on lexical retrieval of content words in sentences in persons with aphasia. Aphasiology 23, 402–424. 10.1080/02687030802291339 19763227 PMC2744980

[B38] Eggert G. H. (1977). Wernicke’s Works in Aphasia: A Sourcebook and Review. Volume 1. The Hague: Mouton.

[B39] Eichenbaum H. (2013). Memory on time. Trends Cogn. Sci. 17, 81–88. 10.1016/j.tics.2012.12.007 23318095 PMC3558533

[B40] Ellis A. Lambon Ralph M. A. (2000). Age of acquisition effects in adult lexical processing reflect loss of plasticity in maturing systems: insights from connectionist networks. J. Exp. Psychol. Learn. Mem. Cogn. 26, 1103–1123. 10.1037/0278-7393.26.5.1103 11009247

[B41] Elman J. L. Bates E. A. Johnson M. H. Karmiloff-Smith A. Parisi D. Plunkett K. (1996). Rethinking Innateness. A Connectionist Perspective on Development. Cambridge, MA: MIT Press.

[B42] Farah M. J. McClelland J. L. (1991). A computational model of semantic memory impairment: modality-specificity and emergent category-specificity. J. Exp. Psychol. Gen. 120, 339–357. 10.1037/0096-3445.120.4.339 1837294

[B43] Farah M. J. O’Reilly R. C. Vecera S. P. (1993). Dissociated overt and covert recognition as an emergent property of a lesioned neural network. Psychol. Rev. 100, 571–588. 10.1037/0033-295x.100.4.571 8255950

[B44] Felleman D. J. Van Essen D. C. (1991). Distributed hiedrarchical processing in the primate cerebral cortex. Cereb. Cortex 1, 1–47. 10.1093/cercor/1.1.1 1822724

[B45] Feng S. F. Schwemmer M. Gershman S. J. Cohen J. D. (2014). Multitasking versus multiplexing: toward a normative account of limitations in the simultaneous execution of control-demanding behaviors. Cogn. Affect. Behav. Neurosci. 14, 129–146. 10.3758/s13415-013-0236-9 24481850 PMC4845905

[B46] Ferretti T. R. McRae K. Hatherell A. (2001). Integrating verbs, situation schemas and thematic role concepts. J. Mem. Lang. 44, 516–547. 10.1006/jmla.2000.2728

[B47] Fiore V. G. Dolan R. J. Strausfeld N. J. Hirth F. (2015). Evolutionarily conserved mechanisms for the selection and maintenance of behavioral activity. Philos. Trans. R. Soc. Lond. B Biol. Sci. 370:20150053. 10.1098/rstb.2015.0053 26554043 PMC4650127

[B48] Forde E. M. E. Humphreys G. W. (1999). Category specific recognition impairments: a review of important case studies and influential theories. Aphasiology 13, 169–193. 10.1080/026870399402172

[B49] Freud E. Plaut D. C. Behrman M. (2016). What is happening in the dorsal visual pathway. Trends Cogn. Sci. 20, 773–784. 10.1016/j.tics.2016.08.003 27615805

[B50] Frewen P. A. Dozois D. J. A. Joanisse M. F. Neufeld R. W. J. (2008). Selective attention to threat versus reward: meta-analysis and neural-network modeling of the dot-probe task. Clin. Psychol. Rev. 28, 307–337. 10.1016/j.cpr.2007.05.006 17618023

[B51] Fries P. (2015). Rhythms for cognition: communication through coherence. Neuron 88, 220–235. 10.1016/j.neuron.2015.09.034 26447583 PMC4605134

[B52] Georgopoulos A. P. Kalaska J. F. Caminiti R. Massey J. T. (1982). On the relations between the direction of two-dimensional arm movements and cell discharge in primate motor cortex. J. Neurosci. 2, 1527–1537. 10.1523/jneurosci.02-11-01527.1982 7143039 PMC6564361

[B53] Geschwind N. (1965). Disconnexion syndromes in animals and man. Brain 88, 237–294. 10.1093/brain/88.2.237 5318481

[B54] Gilbert S. J. Shallice T. (2002). Task switching: a PDP model. Cogn. Psychol. 44, 297–337. 10.1006/cogp.2001.0770 11971634

[B55] Gilmour G. C. Wenk H. E. Naylor L. A. Koss E. (1994). Motor perception and Alzheimer’s disease. J Gerontol: Psychol Sci 49, 52–57. 10.1016/j.jalz.2014.04.514 8126359

[B56] Gleick J. (1987). Chaos: Making a New Science. New York, NY: Viking.

[B57] Glenberg A. M. (1979). Component-levels theory of the effects of spacing of repetitions on recall and recognition. Mem. Cogn. 7, 95–112. 10.3758/bf03197590 459836

[B58] Glenberg A. M. Lehmann T. S. (1980). Spacing repetitions over 1 week. Mem. Cogn. 8, 528–538. 10.3758/bf03213772 7219173

[B59] Goodale M. A. Milner D. (1992). Separate visual pathways for perception and action. Trends Neurosci. 15, 20–25. 10.1016/0166-2236(92)90344-8 1374953

[B60] Grossman M. Ash S. (2004). Primary progressive aphasia: a review. Neurocase 10, 3–18. 10.1080/13554790490960440 15849155

[B61] Gutiérrez R. (2003). The GABAergic phenotype of the “glutamatergic” granule cells of the dentate gyrus. Prog. Neurobiol. 71, 337–358. 10.1016/j.pneurobio.2003.11.004 14757115

[B62] Haberly L. B. (2001). Parallel-distributed processing in olfactory cortex: new insights from morphologivsl and physiological analysis of neuronal circuitry. Chem. Senses 26, 551–576. 10.1093/chemse/26.5.551 11418502

[B63] Hare M. Elman J. L. (1992). “Connectionist account of English infectional morphology: evidence from language change,” in Proceedings of the Fourteenth Annual Conference of the Cognitive Science Society, (Hillsdale, NJ: Erlbaum), 265–270.

[B64] Heilman K. M. (2005). Creativity and the Brain. New York, NY: Taylor and Francis Group.

[B65] Heilman K. M. Nadeau S. E. Beversdorf D. Q. (2003). Creative innovation: possible brain mechanisms. Neurocase 9, 369–379. 10.1076/neur.9.5.369.16553 14972752

[B66] Hélie S. Sun R. (2010). Incubation, insight and creative problem solving: a unified theory and a connectionist model. Psychol. Rev. 117, 994–1024. 10.1037/a0019532 20658861

[B67] Henderson C. M. McClelland J. L. (2011). A PDP model of the simultaneous perception of multiple objects. Connect. Sci. 23, 161–172. 10.1080/09540091.2011.575931

[B68] Hovius M. Kellenbach M. L. Graham K. S. Hodges J. R. Patterson K. (2003). what does the object decision task measure? Reflections on the basis of evidence from semantic dementia. Neuropsychology 17, 100–107. 10.1037/0894-4105.17.1.100 12597078

[B69] Hultén A. Schoffelen J. M. Uddén J. Lam N. H. L. (2019). How the brain makes sense behond the processing of single words — an MEG study. NeuroImage 186, 586–594. 10.1016/j.neuroimage.2018.11.035 30481591

[B70] Iadarola M. J. Berman K. F. Zeffiro T. A. Byas-Smith M. G. Gracely R. H. Max M. B.. (1998). Neural activation during acute capsaicin-evoked pain and allodynia assessed with PET. Brain 121, 931–947. 10.1093/brain/121.5.931 9619195

[B71] Joanisse M. F. McClelland J. L. (2015). Connectionist perspectives on language learning, representation and processing. Wiley Interdiscip. Rev. Cogn. Sci. 6, 235–247. 10.1002/wcs.1340 26263227

[B72] Kahneman D. (2011). Thinking, Fast and Slow. New York, NY: Farrar, Straus and Giroux.

[B73] Kalénine S. Buxbaum L. J. Coslett H. B. (2010). Critical brain regions for action recognition: lesion symptom mapping in left hemisphere stroke. Brain 133, 3269–3280. 10.1093/brain/awq210 20805101 PMC2965423

[B74] Kanne S. M. Balota D. A. Spieler D. H. Faust M. E. (1998). Explorations of Cohen, Dunbar and McClelland’s (1990) connectionist model of Stroop performance. Psychol. Rev. 105, 174–187. 10.1037/0033-295x.105.1.174 9450376

[B75] Karten H. J. (2015). Vertebrate brains and evolutionary connectomics: on the origins of the mammalian ‘neocortex’. Philos. Trans. R. Soc. Lond. B Biol. Sci. 370:20150060. 10.1098/rstb.2015.0060 26554047 PMC4650131

[B77] Kemmerer D. Castillo Gonzalez J. Talavage T. Patterson S. Wiley C. (2008). Neuronanatomical distribution of five semantic components of verbs: evidence from fMRI. Brain Lang. 107, 16–43. 10.1016/j.bandl.2007.09.003 17977592

[B76] Kemmerer D. Gonzalez-Castillo J. (2010). The two-level theory of verb meaning: an approach to integrating the semantics of action with the mirror neuron system. Brain Lang. 112, 54–76. 10.1016/j.bandl.2008.09.010 18996582 PMC2859696

[B79] Kendall D. L. Oelke M. Brookshire C. E. Nadeau S. E. (2015). The influence of phonomotor treatment on word retrieval abilities in 26 individuals with chronic aphasia: an open trial. J. Speech Lang. Hear. Res. 58, 798–812. 10.1044/2015_jslhr-l-14-0131 25766309

[B78] Kendall D. L. Rosenbek J. C. Heilman K. M. Conway T. W. Klenberg K. Gonzalez Rothi L. J.. (2008). Phoneme-based rehabilitation of anomia in aphasia. Brain Lang. 105, 1–17. 10.1016/j.bandl.2007.11.007 18237773

[B80] Kumaran D. Hassabis D. McClelland J. L. (2016). What learning systems do intelligent agents need? Complementary learning systems theory updated. Trends Cogn. Sci. 20, 512–534. 10.1016/j.tics.2016.05.004 27315762

[B81] Lambon Ralph M. A. Ehsan S. (2006). Age of acquisition effects depend on the mapping between representations and the frequency of occurrence: empirical and computational evidence. Visual Cogn. 13, 928–948. 10.1080/13506280544000110

[B84] Lambon Ralph M. A. Ehsan S. Baker G. A. Rogers T. T. (2012). Semantic memory is impaired in patients with unilateral anterior temporal lobe resection for temporal lobe epilepsy. Brain 135, 242–258. 10.1093/brain/awr325 22287382 PMC3267985

[B83] Lambon Ralph M. A. Graham K. S. Patterson K. (1999). Is a picture worth a thousand words? Evidence from concept definitions by patients with semantic dementia. Brain Lang. 70, 309–335. 10.1006/brln.1999.2143 10600223

[B82] Lambon Ralph M. A. Jefferies E. Patterson K. (2017). The neural and computational bases of semantic cognition. Nat. Rev. Neurosci. 18, 42–55. 10.1038/nrn.2016.150 27881854

[B85] Lebedev M. A. Nicolelis M. A. (2017). Brain-machine interfaces: from basic science to neuroprostheses and neurorehabilitation. Physiol. Rev. 97, 767–837. 10.1152/physrev.00027.2016 28275048

[B86] Lissauer H. (1988). Ein fall von seelenblindheit nebst einem beitrag sur theorie derselven. Cogn. Neuropsychol. 5, 157–192.

[B87] Luckman A. J. Alllinson N. M. Ellis A. W. Flude B. M. (1995). Familiar face recognition: a comparative study of a connectionist model and human performance. Neurocomputing 7, 3–27. 10.1016/0925-2312(93)e0052-f

[B88] Marr D. (1971). Simple memory: a theory for archicortex. Philos. Trans. R. Soc. Lond. B Biol. Sci. 262, 23–81. 10.1098/rstb.1971.0078 4399412

[B89] Marshall J. Chiat S. Robson J. Pring T. (1996). Calling a salad a federation: an investigation of semantic jargon. Part 2—verbs. J. Neurolinguistics 4, 251–260. 10.1016/s0911-6044(97)82797-2

[B90] Mata M. Fink D. J. Gainer H. Smith C. B. Davidsen L. Savaki H.. (1980). Activity-dependent energy metabolism in rat posterior pituitary primarily reflects sodium pump activity. J. Neurochem. 34, 213–215. 10.1111/j.1471-4159.1980.tb04643.x 7452237

[B91] Mathews G. Harley T. A. (1996). Connectionist models of emotional distress and attentional bias. Cogn. Emotion 10, 561–600. 10.1080/026999396380060

[B92] McClelland J. L. (2013). Incorporating rapid neocortical learning of new schema-consistent information into complementary learning systems theory. J. Exp. Psychol. Gen. 142, 1190–1210. 10.1037/a0033812 23978185

[B93] McClelland J. L. McNaughton B. L. O’Reilly R. C. (1995). Why there are complementary learning systems in the hippocampus and neocortex: insights from the successes and failures of connectionist models of learning and memory. Psychol. Rev. 102, 419–457. 10.1037/0033-295x.102.3.419 7624455

[B94] McClelland J. L. Mirman D. Bolger D. J. Khaitan P. (2014). Interactive activation and mutual constraint satisfaction in perception and cognition. Cogn. Sci. 38, 1139–1189. 10.1111/cogs.12146 25098813

[B96] McClelland J. L. Rumelhart D. E. PDP Research Group (1986). Parallel Distributed Processing. Cambridge, MA: MIT Press.

[B97] McCloskey M. Cohen N. J. (1989). “Catastrophic interference in connectionist networks: the sequential learning problem,” in The Psychology of Learning and Motivation, ed. Bower G. H. (San Diego, CA: Academic Press), 109–165.

[B98] McRae K. Hare M. Elman J. L. Ferretti T. R. (2005). A basis for generating expectancies for verbs from nouns. Mem. Cogn. 33, 1174–1184. 10.3758/bf03193221 16532852

[B99] Mennemeier M. Pierce C. A. Chatterjee A. Anderson B. Jewell G. Dowler R.. (2005). Biases in attentional orientation and magnitude estimation explain crossover: neglect is a disorder of both. J. Cogn. Neurosci. 17, 1194–1211. 10.1162/0898929055002454 16197678 PMC4442679

[B100] Miceli G. Capasso R. Daaniele A. Esposito T. Magarelli M. Tomaiuolo F. (2000). Selective deficit for people’s names following left temporal damage: an impairment of domain-specific conceptual knowledge. Cogn. Neuropsychol. 17, 489–516. 10.1080/02643290050110629 20945192

[B101] Milner A. D. (2017). How do the two visual streams inrteract with each other? Exp. Brain Res. 235, 1297–1308. 10.1007/s00221-017-4917-4 28255843 PMC5380689

[B102] Milner A. D. Goodale M. A. (2008). Two visual systems re-reviewed. Neuropsychologia 46, 774–785. 10.1016/j.neuropsychologia.2007.10.005 18037456

[B103] Miozzo M. Pulvermüller F. Hauk O. (2015). Early parallel activation of semantics and phonology in picture naming: evidence from a multiple linear regression MEG study. Cereb. Cortex 25, 3343–3355. 10.1093/cercor/bhu137 25005037 PMC4585490

[B104] Mishkin M. Ungerleider L. G. Macko K. A. (1983). Object vision and spatial vision: two cortical pathways. Trends Neurosci. 6, 414–417. 10.1016/0166-2236(83)90190-x

[B105] Monaghan P. Shillcock R. (2004). Hemispheric asymmetries in cognitive modeling: connectionist modeling of unilateral visual neglect. Psychol. Rev. 111, 283–308. 10.1037/0033-295x.111.2.283 15065911

[B106] Moran J. Desimone R. (1985). Selective attention gates visual processing in extrastriate cortex. Science 229, 782–784. 10.1126/science.4023713 4023713

[B107] Moser M. C. Behrmann M. (1990). On the interaction of selective attention and lexical knowledge: a connectionist account of neglect dyslexia. J. Cogn. Neurosci. 2, 96–123. 10.1162/jocn.1990.2.2.96 23972020

[B108] Mozer M. C. (2002). Frames of reference in unilateral neglect and visual perception: a computational perspectiver. Psychol. Rev. 109, 156–185. 10.1037/0033-295x.109.1.156 11863036

[B109] Munakata Y. McClelland J. L. Johnson M. H. Siegler R. S. (1997). Rethinking infant knowledge: toward an adaptive process account of successes and failures in object permanence tasks. Psychol. Rev. 104, 686–713. 10.1037/0033-295x.104.4.686 9337629

[B110] Nadeau S. E. (2010). Hemispheric asymmetry: what, why, and at what cost? J. Int. Neuropsychol. Soc. 16, 593–595. 10.1017/s135561771000041x 20420747

[B111] Nadeau S. E. (2012). The Neural Architecture of Grammar. Cambridge, MA: MIT Press.

[B112] Nadeau S. E. (2014). “Attractor basins: a neural basis for the conformation of knowledge,” in The Roots of Cognitive Neuroscience, eds Chatterjee A. Coslett H. B. (Oxford: Oxford University Press), 305–333.

[B113] Nadeau S. E. (2015). “Neuroplastic mechanisms of language recovery after stroke,” in Cognitive Plasticity in Neurologic Disorders, eds Tracy J. I. Hampstead B. M. Sathian K. (New York, NY: Oxford University Press), 61–84.

[B114] Nadeau S. E. (2019). Bilingual aphasia: explanations in population encoding. J. Neurolinguistics 49, 117–143. 10.1016/j.jneuroling.2018.10.002

[B115] Nadeau S. E. (2020). Basal ganglia and thalamic contributions to language function: insights from a parallel dfistributed processing perspective. Neuropsychol. Rev. 10.1007/s11065-020-09466-0 33512608

[B116] Nadeau S. E. Crosson B. (1997). Subcortical aphasia. Brain Lang. 58, 355–402. 10.1006/brln.1997.1707 9222518

[B117] Norman K. A. (2010). How the hippocampus and cortex contribute to recognition memory: revisiting the complementary learning systems model. Hippocampus 20, 1217–1227. 10.1002/hipo.20855 20857486 PMC3416886

[B118] O’Reilly R. C. Bhattacharyya R. Howard M. D. Ketz N. (2014). Complementary learning systems. Cogn. Sci. 38, 1229–1248. 10.1111/j.1551-6709.2011.01214.x 22141588

[B119] O’Keefe J. Nadel L. (1979). The hippocampus as a cognitive map. Behav. Brain Sci. 2, 487–533. 10.1017/S0140525X00063949

[B120] Peyron R. García-Larrea L. Grégoire M.-C. Costes N. Convers P. Lavenne F.. (1999). Haemodynamic brain responses to acute pain in humans: sensory and attentional networks. Brain 122, 1765–1780. 10.1093/brain/122.9.1765 10468515

[B121] Piaget J. (1936). Origins of Intelligence in the Child. London: Routledge and Kegan Paul.

[B122] Plaut D. C. (1996). Relearning after damage in connectionist networks: toward a theory of rehabilitation. Brain Lang. 52, 25–82. 10.1006/brln.1996.0004 8741976

[B127] Plaut D. C. McClelland J. L. Seidenberg M. S. Patterson K. (1996). Understanding normal and impaired word reading: computational principles in quasi-regular domains. Psychol. Rev. 103, 56–115. 10.1037/0033-295x.103.1.56 8650300

[B124] Plaut D. C. Shallice T. (1991). “Effects of word abstractness in a connectionist model of deep dyslexia,” in Proceedings of the Thirteenth Annual Conference of the Cognitive Science Society, (Hillsdale, NJ: Erlbaum), 73–78.

[B125] Plaut D. C. Shallice T. (1993a). Deep dyslexia: a case study of connectionist neuropsychology. Cogn. Neuropsychol. 10, 377–500. 10.1080/02643299308253469

[B126] Plaut D. C. Shallice T. (1993b). Perseverative and semantic influences on visual object naming errors in optic aphasia: a connectionist account. J. Cogn. Neurosci. 5, 89–117. 10.1162/jocn.1993.5.1.89 23972122

[B123] Plaut D. C. Vande Velde A. K. (2017). Statistical learning of parts and wholes: a neural network approach. J. Exp. Psychol. Gen. 146, 318–336. 10.1037/xge0000262 28080124

[B128] Porter R. Lemon R. (1993). Corticospinal Function and Voluntary Movement. Monographs of the Physiological Society. Oxford: Clarendon Press.

[B129] Prevete R. Tessitore G. Santoro M. Catanzariti E. (2008). A connectionist architecture for view-independent grip-aperture computation. Brain Res. 1225, 133–145. 10.1016/j.brainres.2008.04.076 18538746

[B130] Pulvermüller F. (2010). Brain embodiment of syntax and grammar: discrete combinatorial mechanisms spelt out in neural circuits. Brain Lang. 112, 167–179. 10.1016/j.bandl.2009.08.002 20132977

[B131] Rajkowska G. Goldman-Rakic P. S. (1995). Cytoarchitectonic definition of prefrontal areas in the normal human cortex: II. Variability in locations of areas 9 and 46 and relationship to the Talairach coordinate system. Cereb. Cortex 5, 323–337. 10.1093/cercor/5.4.323 7580125

[B132] Ranganath C. Hsieh L.-T. (2016). The hippocampus: a special place for time. Ann. N Y Acad. Sci. 1369, 93–110. 10.1111/nyas.13043 27082833

[B133] Raposo A. Moss H. E. Stamatakis E. A. Tyler L. K. (2009). Modulation of motor and premotor cortices by actions, action words and action sentences. Neuropsychologia 47, 388–396. 10.1016/j.neuropsychologia.2008.09.017 18930749

[B134] Reinhart R. M. G. Nguyen J. A. (2019). Working memory revived in older adults by synchronizing rhythmic brain circuits. Nat. Neurosci. 22, 820–827. 10.1038/s41593-019-0371-x 30962628 PMC6486414

[B135] Rogers T. T. Ivanoiu A. Patterson K. Hodges J. R. (2006). Semantic memory in Alzheimer’s disease and the frontotemporal dementias: a longitudinal study of 236 patients. Neuropsychology 20, 319–335. 10.1037/0894-4105.20.3.319 16719625

[B140] Rogers T. T. Lambon Ralph M. A. Garrard P. Bozeat S. McClelland J. L. Hodges J. R.. (2004). Structure and deterioration of semantic memory: a neuropsychological and computational investigation. Psychol. Rev. 111, 205–235. 10.1037/0033-295x.111.1.205 14756594

[B136] Rogers T. T. McClelland J. L. (2004). Semantic Cognition. A Parallel Distributed Processing Approach. Cambridge, MA: MIT Press. 10.1038/nrn1076 12671647

[B137] Rogers T. T. McClelland J. L. (2008). Précis of semantic cognition: a parallel distributed processing approach. Behav. Brain Sci. 31, 689–748. 10.1017/S0140525X0800589X

[B138] Rogers T. T. McClelland J. L. (2014). Parallel distributed processing at 25: further explorations in the microstructure of cognition. Cogn. Sci. 38, 1025–1077. 10.1111/cogs.12148 25087578

[B139] Rogers T. T. Patterson K. Jefferies E. Ralph M. A. (2015). Disorders of representation and control in semantic cognition: effects of familiarity, typicality, and specificity. Neuropsychologia 76, 220–239. 10.1016/j.neuropsychologia.2015.04.015 25934635 PMC4582808

[B141] Rolls E. T. (2016). Cerebral Cortex: Principles of Operation. Oxford: Oxford University Press.

[B143] Rolls E. T. Deco G. (2002). Computational Neuroscience of Vision. Oxford: Oxford University Press.

[B144] Rolls E. T. Deco G. (2015). Stochastic cortical neurodynamics underlying the memory and cognitive changes in aging. Neurobiol. Learn. Mem. 118, 150–161. 10.1016/j.nlm.2014.12.003 25536108

[B145] Roth H. L. Nadeau S. E. Hollingsworth A. L. Marie Cimino-Knight A. Heilman K. M. (2006). Naming concepts: evidence of two routes. Neurocase 12, 61–70. 10.1080/13554790500502892 16517516

[B142] Rolls E. T. Treves A. (1998). Neural Networks and Brain Function. New York, NY: Oxford University Press.

[B146] Schapiro A. C. McClelland J. L. (2009). A connectionist model of a continuous developmental transition in the balance scale task. Cognition 110, 395–411. 10.1016/j.cognition.2008.11.017 19171326

[B147] Schwartz W. J. Smith C. B. Davidsen L. Savaki H. Sokoloff L. Mata M.. (1979). Metabolic mapping of functional activity in the hypothalamo-neurohypophysial system of the rat. Science 205, 723–725. 10.1126/science.462184 462184

[B149] Seidenberg M. S. McClelland J. L. (1989). A distributed, developmental model of word recognition and naming. Psychol. Rev. 96, 523–568. 10.1037/0033-295x.96.4.523 2798649

[B148] Seidenberg M. S. Plaut D. C. (2014). Quasiregularity and its discontents: the legacy of the past tense debate. Cogn. Sci. 38, 1190–1228. 10.1111/cogs.12147 25104139

[B150] Spalding K. L. Bergmann O. Alkass K. Bernard S. Salehpour M. Huttner H. B.. (2013). Dynamics of hippocampal neurogenesis in adult humans. Cell 153, 1219–1227. 10.1016/j.cell.2013.05.002 23746839 PMC4394608

[B151] Spratling M. W. Johnson M. H. (2004). A feedback model of visual attention. J. Cogn. Neurosci. 16, 219–237. 10.1162/089892904322984526 15068593

[B152] Squire L. R. Zola-Morgan S. (1991). The medial temporal lobe memory system. Science 253, 1380–1386. 10.1126/science.1896849 1896849

[B153] Stevens S. S. (1957). On the psychophysical law. Psychol. Rev. 64, 153–181. 10.1037/h0046162 13441853

[B154] Stevens S. S. (1970). Neural events and the psychophysical law. Science 170, 1043–1050. 10.1126/science.170.3962.1043 5475633

[B155] Tang H. Schrimpf M. Lotter W. Moerman C. Paredes A. Ortega Caro J.. (2018). Recurrent computations for visual pattern completion. Proc. Natl. Acad. Sci. U S A 115, 8835–8840. 10.1073/pnas.1719397115 30104363 PMC6126774

[B156] Tegnér R. Levander M. (1991). The influence of stimulus properties on visual neglect. J. Neurol. Neurosurg. Psychiatry 54, 882–887. 10.1136/jnnp.54.10.882 1744642 PMC1014572

[B157] Tononi G. Cirelli C. (2014). Sleep and the price of plasticity: from synaptic and cellular homeostasis to memory consolidation and integration. Neuron 81, 12–34. 10.1016/j.neuron.2013.12.025 24411729 PMC3921176

[B158] Tranel D. Manzel K. Asp E. Kemmerer D. (2008). Naming dynamic and static actions: neuropsychological evidence. J. Physiol. 102, 80–94. 10.1016/j.jphysparis.2008.03.008 18486456 PMC2519898

[B159] Tressoldi P. E. Sella F. Coltheart M. Umiltå C. (2012). Using functional neuroimaging to test theories of cognitioin: a selective survey of studies from 2007 to 2011 as a contribution to the Decade of the Mind Initiative. Cortex 48, 1247–1250. 10.1016/j.cortex.2012.05.024 22795266

[B160] Tse D. Langston R. F. Kakeyama M. Bethus I. Spooner P. A. Wood E. R.. (2007). Schemas and memory consolidation. Science 316, 76–82. 10.1126/science.1135935 17412951

[B161] Tse D. Takeuchi T. Kakeyama M. Kajii Y. Okuno H. Tohyama C.. (2011). Schema-dependent gene activation and memory encoding in neocortex. Science 333, 891–895. 10.1126/science.1205274 21737703

[B162] Vargha-Khadem F. Gadian D. G. Watkins K. E. Connelly A. Van Paesschen W. Mishkin M. (1997). Differential effects of early hippocampal pathology on episodic and semantic memory. Science 277, 376–380. 10.1126/science.277.5324.376 9219696

[B163] Vitevitch M. S. Castro N. (2015). Using network science in the language sciences and clinic. Int. J. Speech Lang. Pathol. 17, 13–25. 10.3109/17549507.2014.987819 25539473 PMC5609822

[B164] Vitevitch M. S. Luce P. A. (2016). Phonological neighborhood effects in spoken word perception and production. Annu. Rev. Linguist. 2, 75–94. 10.1146/annurev-linguistics-030514-124832

[B165] Warrington E. K. Shallice T. (1984). Category specific semantic impairments. Brain 107, 829–854. 10.1093/brain/107.3.829 6206910

[B166] Winocur G. Moscovitch M. Bontempi B. (2010). Memory formation and long-term retention in humans and animals: convergence towards a transformation account of hippocampal-neocortical interactions. Neuropsychologia 48, 2339–2356. 10.1016/j.neuropsychologia.2010.04.016 20430044

[B167] Woollams A. M. Cooper-Pye E. Hodges J. R. Patterson K. (2008). Anomia: a doubly typical signature of semantic dementia. Neuropsychologia 46, 2503–2514. 10.1016/j.neuropsychologia.2008.04.005 18499196

[B168] Wu D. H. Morganti A. Chatterjee A. (2008). Neural substrates of processing path and manner information of a moving event. Neuropsychologia 46, 704–713. 10.1016/j.neuropsychologia.2007.09.016 18023824 PMC2271062

[B169] Wurtz R. H. (2008). Neuronal mechanisms of visual stability. Vision Res. 48, 2070–2089. 10.1016/j.visres.2008.03.021 18513781 PMC2556215

[B170] Yoon E. Y. Heinke D. Humphreys G. W. (2002). Modelling direct perceptual constraints on action selection: the Naming and Action Model (NAM). Visual Cogn. 9, 615–661. 10.1080/13506280143000601

[B171] Zachariou V. Nikas C. V. Safiullah Z. N. Behrmann M. Klatzky R. Ungerleider L. G. (2015). Common dorsal stream substrates for the mapping of surface texture to object parts and visual spatial processing. J. Cogn. Neurosci. 27, 2442–2461. 10.1162/jocn_a_00871 26359538 PMC6632085

[B172] Zachariou V. Nikas C. V. Safiullah Z. N. Gotts S. J. Ungerleider L. G. (2017). Spatal mechanisms within the dorsal visual pathway contribute to the configural processing of faces. Cereb. Cortex 27, 4124–4138. 10.1093/cercor/bhw224 27522076 PMC6248673

[B173] Zebrowitz L. A. Kikuchi M. Fellous J.-M. (2010). Facial resemblance to emotions: group differences, impression effects, and race stereotypes. J. Pers. Soc. Psychol. 98, 175–189. 10.1037/a0017990 20085393 PMC3677560

[B175] Zhang K. Ginzburg I. McNaughton B. L. Sejnowski T. J. (1998). Interpreting neuronal population activity by reconstruction: unified framework with application to hippocampal place cells. J. Neurophysiol. 79, 1017–1044. 10.1152/jn.1998.79.2.1017 9463459

[B174] Zhang K. Sejnowski T. J. (1999). Neuronal tuning: to sharpen or broaden? Neural Comput. 11, 75–84. 10.1162/089976699300016809 9950722

